# Comparative effects of biventricular and right ventricular pacing on clinical outcomes in atrioventricular block: a systematic review and meta-analysis

**DOI:** 10.1186/s12872-025-05336-w

**Published:** 2025-11-25

**Authors:** Ayan Khalid, Anas Rasool, Shaikh Muhammad Daniyal, Muhammad Ibrahim, Fauzaan Ahmed Siddiqui, Muhammad Saad, Muhammad Hasan Siddiqui, Rahul Balach, Hafiz Bilal Ahmed, Isbah Gul, Yamaan Adil, Sabula Tabish, Danish Ali Ashraf, Ahmed Sajid, Romal Jabarkhil

**Affiliations:** 1 Department of Medicine, Dow University Of Health Sciences, Karachi, Pakistan; 2 Department of Medicine, Ziauddin University, Karachi, Pakistan; 3 Department of Medicine, TruGift Health LLC, Wilmington, DE USA; 4 Department of Medicine, Muzaffar Khan Mother and Children Hospital, Islamabad, Pakistan; 5 Department of Medicine, Ningarhar Regional Hospital, Jalalabad, Afghanistan

**Keywords:** Electrophysiology, Mortality, Resynchronization therapy, Biventricular pacing

## Abstract

**Background:**

Right ventricular (RV) pacing is the standard treatment for atrioventricular (AV) block but may impair left ventricular (LV) function over time. Biventricular (BiV) pacing offers a physiologic alternative, though prior evidence has been mixed, particularly after the BioPace trial.

**Methods:**

PubMed, ScienceDirect, Google Scholar, and the Cochrane Library were searched through May 2025 for randomized controlled trials (RCTs) comparing BiV and RV pacing in AV block. Thirteen RCTs (*n* = 3,685) met the inclusion criteria. Outcomes included heart failure (HF) hospitalization, all-cause mortality, six-minute walk distance (6MWD), cardiovascular (CV) death, and LVEF (%) at follow-up.

**Results:**

BiV pacing significantly reduced HF hospitalizations (risk ratio [RR] = 0.83, 95% CI 0.69–0.98; *p* = 0.03) and better preserved LVEF (mean difference = 6.17%, 95% CI 3.85–8.49; *p* < 0.00001). No significant differences were observed in all-cause mortality (RR = 0.87, 95% CI 0.70–1.08; *p* = 0.21) or CV death (RR = 0.95, 95% CI 0.75–1.21; *p* = 0.70). RV pacing showed a borderline improvement in 6MWD (mean difference = 10.3 m, 95% CI − 0.1 to 20.7; *p* = 0.05).

**Conclusion:**

In patients with AV block and high pacing burden, BiV pacing reduced HF hospitalizations and better preserved systolic function compared with RV pacing. These findings support a burden-based pacing strategy, while CRT use should be individualized, considering patient profile and procedural factors.

**Systematic review registration:**

This systematic review and meta-analysis was retrospectively registered in the International Prospective Register of Systematic Reviews (PROSPERO) under ID 1080380.

**Clinical trial number:**

Not applicable.

**Supplementary Information:**

The online version contains supplementary material available at 10.1186/s12872-025-05336-w.

## Introduction

Atrioventricular (AV) block is one of the leading causes of pacemaker implants [1]. Ventricular pacing (VP) is a vital treatment for patients with AV block, with right ventricular (RV) pacing being the traditional approach [2]. However, current evidence suggests that chronic RV pacing may lead to electrical and mechanical dyssynchrony, causing adverse left ventricular (LV) remodeling, heart failure (HF), and increased mortality [3, 4]. 

For instance, a study by Sohn et al. reported that mortality and HF-related hospitalization occurred in 8.1% and 17% of patients, respectively, after chronic RV pacing for acquired AV block [5]. Similarly, in a trial by Sweeney et al., patients with >80% RV pacing burden had a 3.58 times higher risk of HF hospitalization compared to those with less pacing [6].

To counter the adverse effects, Biventricular (BiV) pacing, a component of cardiac resynchronization therapy (CRT), is increasingly considered for patients with AV block. CRT replicates the physiological interventricular conduction properties and improves LV function and clinical outcomes in patients with heart failure and prolonged QRS duration [7–13].

In a study done by Lu et al., BiV Pacing significantly lowered all-cause mortality and HF hospitalization rates compared to RV pacing in patients with AV block who require frequent VP [14]. Additionally, BiV Pacing has been linked with reducing morbidity and adverse LV remodeling in patients with AV block and LV systolic dysfunction [2]. It has also been shown to minimize LV dyssynchrony and preserve LV function, likely by restoring coordinated activation of both ventricles and improving mechanical contraction [15].

Several studies have compared the effects of the BiV and RV pacing modalities in patients with high-grade AV block or AV junction (AVJ) ablation-induced AV block [2, 16–18]. However, these studies are limited by small sample sizes and conflicting clinical outcomes. Although randomized controlled trials (RCTs) have also explored the effects of the two modalities, but findings remain inconsistent across key outcomes, including mortality, HF hospitalization, and functional capacity as measured by the 6-minute walk distance (6MWD). Notably, the divergence between previous meta-analyses and the recent published data highlights the need for an updated and comprehensive evaluation of the clinical outcomes associated with BiV pacing. To address this, we conducted a systematic review and meta-analysis (SRMA) of RCTs comparing BiV pacing with RV pacing in patients requiring frequent VP.

## Methods

This SRMA was conducted in strict adherence to the Cochrane Handbook for Systematic Reviews of Interventions and the Preferred Reporting Items for Systematic Reviews and Meta-Analyses (PRISMA) guidelines [19, 20]. The study protocol was pre-registered on the International Prospective Register of Systematic Reviews (PROSPERO) under ID 1,080,380.

### Search strategy and selection criteria

PubMed, ScienceDirect, Google Scholar, and the Cochrane Library were systematically searched for all RCTs comparing BiV pacing with RV pacing in patients with AV block, including those with AVJ ablation-induced AV block, from inception to May 2025. The search strategy utilized for each database is detailed in Supplementary Table 1. All articles retrieved through the systematic search were exported to Rayyan.ai, and duplicates were removed. The remaining articles were evaluated by two independent reviewers, followed by a full-text review to assess their applicability. Any discrepancies were resolved through discussion until a consensus was reached.

The inclusion criteria were defined in accordance with the PICOT framework. The study population comprised adults with AV block, either intrinsic or secondary to AVJ ablation, requiring VP. The intervention of interest was BiV pacing or CRT, compared with conventional RV pacing. Eligible studies were required to report at least one outcome of interest, including all-cause mortality, HF hospitalization, 6MWD, cardiovascular (CV) death, or LVEF (%) at follow-up, with a minimum follow-up duration of at least three months. Studies were included if at least 50% of the enrolled population had AV block, either intrinsic or induced by AVJ ablation.

### Data extraction and outcomes of interest

Two authors independently extracted data from the selected articles, resolving any conflicts through discussion until consensus was reached. In addition to baseline and patient characteristics, data were gathered on the prespecified outcomes, specifically comparing outcomes with RV pacing in patients with AV block. The Cochrane Risk of Bias 2.0 (RoB 2.0) Tool was utilized to assess the risk of bias in the RCTs [21].

### Statistical analysis

For continuous outcomes, mean differences (MDs) were analyzed using the inverse variance method. For dichotomous outcomes, analyses were conducted on the log risk ratio (log RR) scale using the Cochran-Mantel-Haenszel method. A random-effects model was utilized to compute all associated 95% confidence intervals (CIs). Heterogeneity was assessed using the I² statistic, with values ≤ 50% considered acceptable.

Forest plots were generated using Review Manager (version 5.4; The Cochrane Collaboration, Copenhagen). Funnel plots, Egger’s test, and meta-regression were conducted using Comprehensive Meta-Analysis (version 4.0; Biostat, Englewood, NJ, USA). Funnel plot asymmetry was visually inspected, and publication bias was evaluated using Egger’s regression.

Subgroup analyses explored the effect of baseline left ventricular ejection fraction (LVEF) (< 50 vs. >50%) on outcomes. Additional analyses were performed to evaluate differences by RV pacing site (apical vs. non-apical), age group (< 70 vs. ≥70 years), and VP burden (permanent pacing vs. high pacing burden > 80%).

## Results

**Fig. 1:**
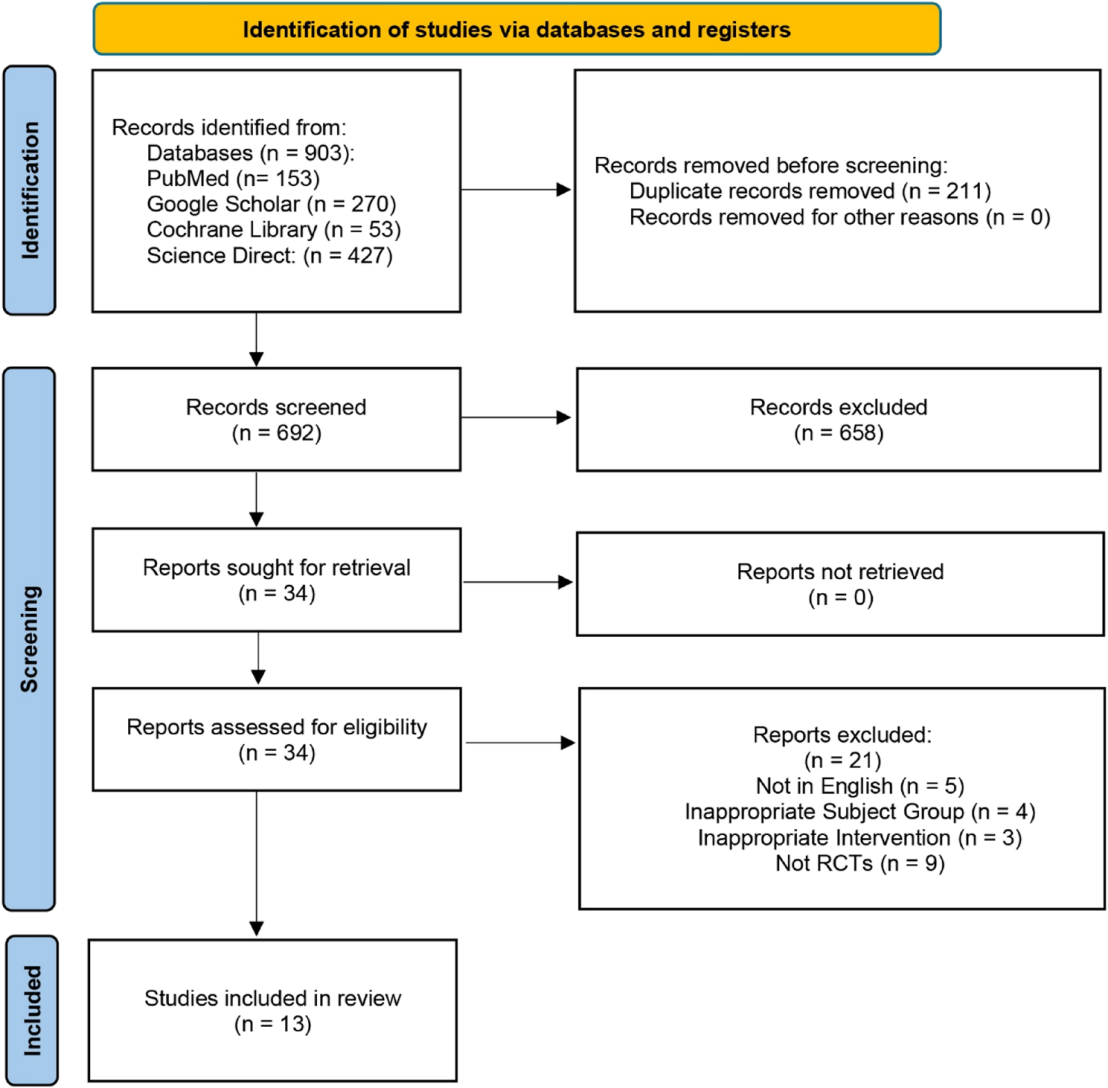
PRISMA flow diagram of the study selection process illustrating the number of records identified, screened, assessed for eligibility, and included in the review

We identified 903 potentially relevant studies, of which 658 studies were excluded after screening of titles and abstracts. Full texts of 34 articles were reviewed, and 21 studies did not meet the inclusion criteria. The analysis is primarily based on the results of the shortlisted 13 studies, all of which are RCTs [2, 10, 13, 15, 16, 18, 22–28]. Where multiple follow-ups were reported, only the longest was used for analysis. The follow-up durations of the included studies ranged from 3 to 68.8 months, with a mean follow-up of approximately 25.0 months. The PRISMA flow chart summarizes the search and trial selection (Fig. 1).

### Baseline characteristics and bias assessment

Table 1 summarizes the baseline characteristics of the included RCTs. Four of these were crossover studies [LECLERCQ, HOBIPACE, COMBAT, OPSITE] [10, 13, 22, 23], while the other nine were parallel-group RCTs. The studies were published between 2002 and 2025. Most trials involved older patients, with the average weighted mean age being 69.7 years. Men made up the majority of participants in all studies, with proportions ranging from 40% to 81.4%. Baseline LVEF varied considerably, from as low as 26% in LECLERCQ to as high as 61.9% in PACE. Baseline VP rates were consistently high: several studies reported VP rates ≥ 97%, some used permanent pacing protocols, and all studies had a mean VP burden exceeding 80%. Several trials, such as LECLERCQ, AVAIL, APAF, OPSITE, and PAVE included patients with atrial fibrillation (AF) who had undergone AVJ ablation and therefore needed continuous VP [16, 18, 22, 23, 28]. Other trials, including HOBIPACE, COMBAT, and BIVPACE-AVB, focused on patients with AV block and HF [10, 13, 24].

**Table 1:** Baseline characteristics of included studies

Study (first author)	Year	Group	VP rate (%)	No.	Age (years)	Male (%)	Baseline BP (mmHg)	Baseline LVEF (%)
Cross-over RCTs MUSTIC (Leclercq)^a^	2002	RVP → BiVP	Permanent	43	65	81.4	NA	26 ± 10
HOBIPACE (Kindermann)	2006	RVP → BiVP	Permanent	30	69.6	77	NA	26.1 ± 7.8
COMBAT (Martinelli)	2010	RVP	NA	31	57.4	67.7	NA	29.2 ± 7.4
		BiVP	NA	29	59.3	62.1	NA	30.1 ± 9.2
OPSITE (Brignole)^a^	2005	RVP → BiVP	Permanent	56	70	61	NA	38.0 ± 14.0
Parallel RCTs PAVE (Doshi)^a^	2005	RVP	Permanent	106	67	64	NA	45.0 ± 15.0
		BiVP	Permanent	146	70	63	NA	47.0 ± 16.0
Albertsen	2008	RVP	100	25	76	68	155/83	59.7 (57–61)
		BiVP	100	25	76	68	146/79	58.9 (47–62)
PACE (Yu, C)	2009	RVP	97	88	68	56	143/69	61.5 ± 6.6
		BiVP	98	89	69	53	148/73	61.9 ± 6.7
APAF (Brignole)^a^	2011	RVP	Permanent	89	72	73	NA	37 ± 14
		BiVP	Permanent	97	72	67	NA	38 ± 14
AVAIL (Orlov)^a^	2010	RVP	97	20	70.1	65	NA	57.2 ± 7.5
		BiVP	98	88	73.0	40	NA	56.1 ± 9.4
PREVENT-HF (Stockburger)	2011	RVP	≥80	58	69.5	76	141.7	54.9 + 12.9
		BiVP	≥80	50	71.6	68	139.1	57.5 + 11.8
BLOCK HF (Curtis)	2013	RVP	≥97	342	73	72.8	NA	39.6 ± 8.3
		BiVP	≥97	349	73.7	76.8	NA	40.3 ± 8.4
BIVPACE-AVB (Zhang)	2016	RVP	95.47	57	66	70.2	126.1/74.4	60.6 ± 9.5
		BiVP	96.32	57	67.1	71.9	125.9/76.4	59.4 ± 10.2
BIOPACE	2025	RVP BiVP	86.4 90.2	908 902	73.3 73.8	67.4 69.2	NA NA	55.5 ± 12.4 55.3 ± 12.1

Data were mean ± SD or median (25th, 75th)

*RCTs* Randomized controlled trials, *RVP* Right Ventricular pacing, *BiVP* Biventricular pacing, *FU* Follow-up, *VP* Ventricular pacing, *No* Number of patients, *BP* Blood pressure, *LVEF* Left ventricular ejection fraction, *NA* Not available

^a^Studies in which patients underwent atrioventricular junction or His bundle ablation

As mentioned in Table 2, the RV apex was the most commonly used pacing site across studies for RV leads. However, some trials used alternative sites: Albertsen used the RV outflow tract, COMBAT used the mid to lower interventricular septum, and in LECLERCQ, they were positioned as far from the left as possible [13, 15, 23]. For LV pacing, leads were usually placed in the lateral or posterolateral branches of the coronary sinus, though some studies, such as APAF and LECLERCQ, used mid-lateral or basal sites instead [18, 23].

**Table 2:** Study population and leads implantation of included trials

Study	Study population	RV pacing position	LV leads placement
HOBIPACE	Patients with symptomatic bradycardia and AVB that required permanent VP; LVDD ≥ 60 mm and EF ≤ 40%	17 RV septum and 13 RV apex	20 lateral, posterior, or posterolateral position and 10 anterolateral position
Albertsen	Patients with permanent or paroxysmal high-grade AVB	RV outflow tract	Lateral or posterolateral branch of the coronary sinus tributary
PACE	Patients with standard indications for pacing, including sinus-node dysfunction and bradycardia due to advanced AVB; EF > 45%	RV apex	Posterolateral or lateral venous branches of the coronary sinus
COMBAT	Patients with AVB, NYHA class II–IV, and LVEF ≤ 40%	The mid or lower portion of the interventricular septal wall	A branch of the coronary sinus over the posterolateral or lateral LV wall
PREVENT-HF	Adults met class I or IIa implantation criteria for pacemaker stimulation and with a high expected need for VP (at least 80%) due to AVB	RV apex	LV lateral wall
BLOCK HF	Patients with a standard indication for VP for AVB, EF ≤ 50%, and mild-to-moderate heart failure	NA	NA
BIVPACE-AVB	Patients with high-grade AVB, NYHA class I–III, and LVEF ≥ 35%	RV apex	Preferentially lateral position
MUSTIC (Leclercq)^a^	NYHA class III, LVEF < 35%, LVDD > 60 mm, and persistent AF needing permanent VP	As far from the left as possible	Preferably mid-lateral
OPSITE^a^	Permanent AF needing AVJ ablation and resistant HF needing CRT	RV apex	Positioned via the coronary sinus or epicardial
PAVE^a^	Chronic AF needing AV nodal ablation, 6MWD < 450 m	RV apex	Via coronary sinus
AVAIL^a^	AF with indication for AV nodal ablation, NYHA class II–III, age ≥ 18 years	NA	NA
APAF^a^	Permanent AF with indications for AVJ ablation, permanent AF with drug-refractory needing CRT.	RV apex	Basal or mid-portion of the postero-lateral free wall
BIOPACE	Patients with preserved LVEF, narrow QRS, and HVPB	70 RV septum, 794 RV apex, 32 other sites, and 12 not implanted	Implanted in the lateral and posterolateral veins

*RV *Right ventricular, *LV * Left ventricular, *AVB * Atrioventricular block, *NYHA * New York Heart Association, *LVDD * LVend-diastolic diameter, *EF * Ejection fraction, *AVJ * Atrioventricular junction, *CRT * Cardiac resynchronization therapy, *AF * Atrial fibrillation, *NA* Not available, *HVPB* High ventricular pacing burden

^a^Studies with AF patients undergoing AVJ ablation

Of the 13 studies, 10 had low risk of bias, 1 (PREVENT-HF) had some concerns, and 2 (APAF and BIVPACE-AVB) had high risk. Most studies were methodologically strong, with few showing notable limitations (as shown in the Supplementary Fig. S1).

### Results of meta-analysis

RRs and MDs were used for reporting data as measures of association for dichotomous and continuous outcomes, respectively.

#### Heart failure hospitalization

**Fig. 2:**
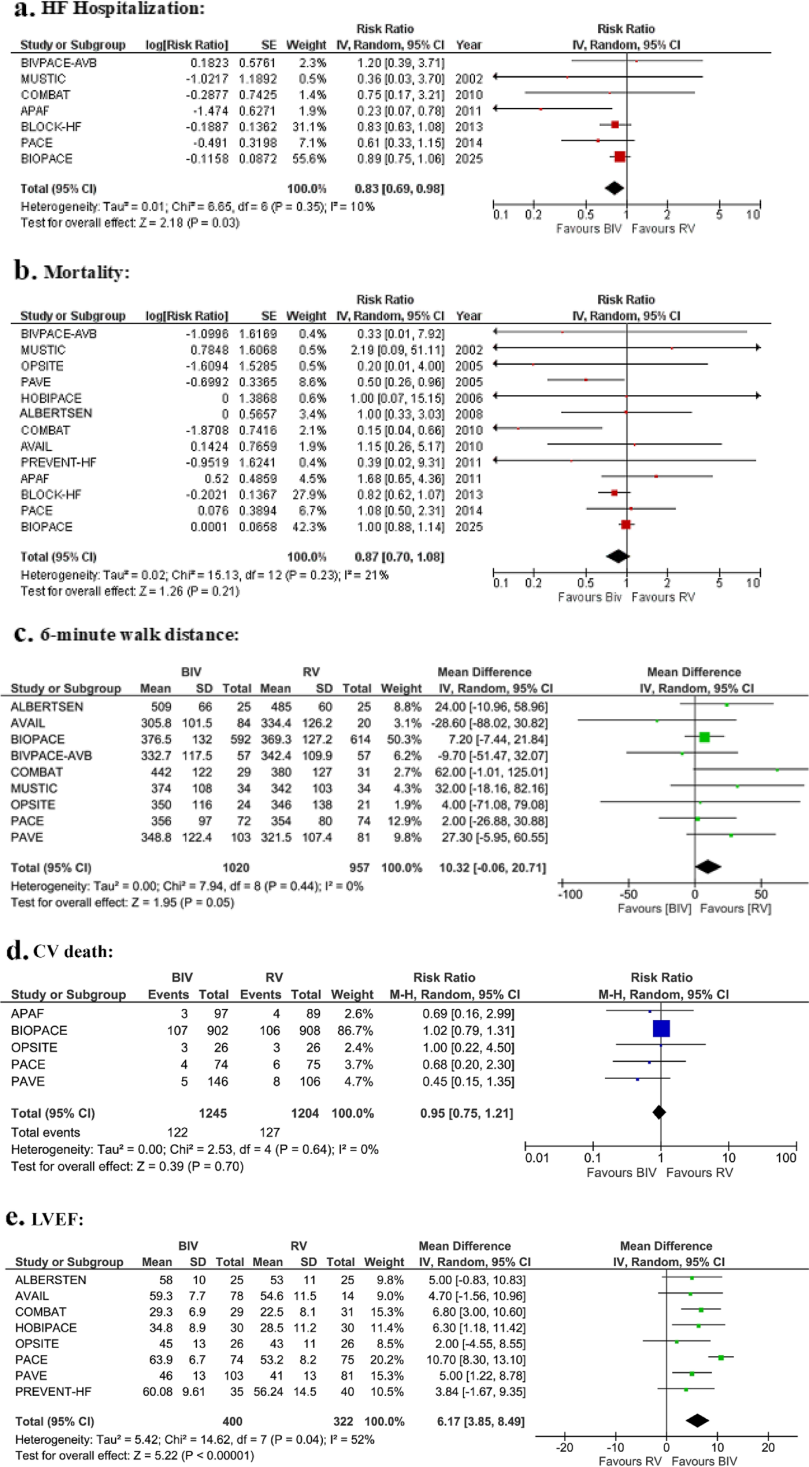
The comparison of BiV pacing with RV pacing on (**a**) HF hospitalization, (**b**) Mortality, (**c**) 6-minute walk distance, (**d**) Cardiovascular death, and (**e**) LVEF (%) in patients with atrioventricular block

Seven of the thirteen included studies assessed the comparative effectiveness of BiV pacing versus RV pacing for HF hospitalization in patients with AV block, including those with AVJ ablation-induced AV block. The pooled analysis demonstrated that BiV pacing was associated with a statistically significant reduction in HF hospitalization compared to RV pacing (RR: 0.83 [95% CI: 0.69 to 0.98]; *p* = 0.03; I² = 10%; Fig. 2a). Subgroup analysis showed no effect modification by baseline LVEF (p-interaction = 0.66; Supplementary Fig. S2a), RV pacing site (p-interaction = 0.94; Supplementary Fig. S3a), or age (p-interaction = 0.63; Supplementary Fig. S4a), but a significant interaction was observed for VP burden (p-interaction = 0.03; Supplementary Fig. S5a). Sensitivity analysis excluding studies involving AVJ ablation-induced AV block eliminated heterogeneity (RR: 0.95 [95% CI: 0.85 to 1.07]; *p* = 0.39; I² = 0%; Supplementary Fig. S6a). Both the funnel plot and Egger’s test (*p* = 0.076) indicated no statistically significant publication bias for HF hospitalization (Supplementary Fig. S7a).

#### All-cause mortality

Thirteen studies evaluated the impact of BiV pacing compared to RV pacing on all-cause mortality. Pooled analysis suggested a trend toward reduced mortality with BiV pacing, although the difference did not reach statistical significance (RR: 0.87 [95% CI: 0.70 to 1.08]; *p* = 0.21; I² = 21%; Fig. 2b). Subgroup analysis showed no significant effect modification by baseline LVEF (p-interaction: 0.34; Supplementary Fig. S2b), RV pacing site (p-interaction = 0.65; Supplementary Fig. S3b), age (p-interaction = 0.13; Supplementary Fig. S4b), or VP burden (p-interaction = 0.35; Supplementary Fig. S5b). Sensitivity analysis excluding studies involving AVJ ablation-induced AV block substantially attenuated heterogeneity (RR: 0.81 [95% CI: 0.64 to 1.03]; *p* = 0.08; I² = 0%; Supplementary Fig. S6b). Both the funnel plot and Egger’s test (*p* = 0.104) indicated no statistically significant publication bias for mortality (Supplementary Fig. S7b).

#### 6-Minute walk distance

Nine studies assessed the impact of BiV pacing versus RV pacing on functional capacity, as measured by the 6MWD. The pooled analysis showed a borderline significant improvement in walking distance with RV pacing compared to BiV pacing (MD: 10.32 m [95% CI: −0.06 to 20.71]; *p* = 0.05; I² = 0%; Fig. 2c). Subgroup analysis showed no significant effect modification by baseline LVEF (p-interaction: 0.28; Supplementary Fig. S2c), RV pacing site (p-interaction = 0.42; Supplementary Fig. S3c), age (p-interaction = 0.49; Supplementary Fig. S4c), or VP burden (p-interaction = 0.08; Supplementary Fig. S5c). Sensitivity analysis excluding studies involving AVJ ablation-induced AV block slightly increased heterogeneity; however, it remained low (MD: 9.22 m [95% CI: −4.08 to 22.51]; *p* = 0.17; I² = 11%; Supplementary Fig. S6c). Both the funnel plot and Egger’s test (*p* = 0.301) indicated no statistically significant publication bias for the 6MWD (Supplementary Fig. S7c).

#### Cardiovascular death

Five studies evaluated the impact of BiV pacing compared to RV pacing on CV death. Pooled analysis suggested a trend toward reduced mortality with BiV pacing, although the difference did not reach statistical significance (RR: 0.95 [95% CI: 0.75 to 1.21]; *p* = 0.70; I² = 0%; Fig. 2d). Subgroup analysis didn’t reveal effect modification by LVEF (p-interaction: 0.90; Supplementary Fig. S2d) and age (p-interaction: 0.89; Supplementary Fig. S4d). Both the funnel plot and Egger’s test (*p* = 0.075) indicated no statistically significant publication bias (Supplementary Fig. S7d).

#### LVEF (%)

Across eight studies, BiV pacing was associated with a significantly higher mean LVEF at the longest available follow-up compared with RV pacing (MD = 6.17 [95% CI 3.85 to 8.49]; *p* < 0.00001; *I²* = 52%; Fig. 2e). Subgroup analysis showed no significant effect modification by baseline LVEF (p-interaction: 0.63; Supplementary Fig. S2e), RV pacing site (p-interaction = 0.91; Supplementary Fig. S3d), age (p-interaction = 0.10; Supplementary Fig. S4e), or VP burden (p-interaction = 0.46; Supplementary Fig. S5d). Sensitivity analysis performed by excluding the PACE study eliminated heterogeneity, while the effect size remained preserved (MD = 5.20 [95% CI 3.34 to 7.06]; *p* < 0.00001; *I²* = 0%; Fig. S6d). The funnel plot and Egger’s test (*p* = 0.00087) indicated significant publication bias (Supplementary Fig. S7e).

## Discussion

In this meta-analysis of 3,685 patients with AV block and high VP burden (> 80%), BiV pacing reduced HF hospitalizations by 17% compared to RV pacing, and was associated with a significantly higher LVEF at the longest available follow-up, indicating better preservation of systolic function over time. BiV pacing showed a non-significant 13% mortality reduction consistent with CRT’s known survival trends. BiV pacing prevents HF hospitalizations by correcting pacing-induced dyssynchrony in AV block patients but shows no mortality benefit as deaths can often be from non-cardiac causes (age, comorbidities) or arrhythmias.

The previous SRMA, Lu et al., reported significantly lower mortality and HF hospitalizations with BiV pacing [14]. Notably, our mortality findings diverge from Lu et al.’s with BiV pacing. This discrepancy likely reflects fundamental differences in the included populations; our study incorporated broader populations, including BioPace’s predominantly preserved LVEF cohort [26]. Despite non-significance, the CIs rule out substantial harm and still allow for a possible clinical benefit. A borderline trend favored RV pacing for 6MWD, but its clinical significance remains uncertain. Lu et al. reported a negligible difference for the 6MWD outcome, though the direction of effect similarly favored RV pacing over BiV pacing [14].

CRT is a well-established treatment for patients with AV block, HF, reduced LVEF, and intraventricular conduction delay, such as left bundle branch block (LBBB). Its main goal is to correct ventricular dyssynchrony, improve HF symptoms, and reduce mortality [29–36].

Among patients with preserved LVEF, evidence demonstrating clear superiority of BiV pacing remains limited. The multicentre BioPace trial, the largest and most statistically influential study included, found no significant differences in mortality or HF hospitalizations between BiV and RV pacing, despite enrolling patients with a high VP burden. The predominantly preserved LVEF and normal QRS duration reflected a population with minimal baseline conduction delay, limiting the potential for meaningful resynchronization benefit [26, 37–39]. Furthermore, a substantial crossover rate, mainly from BiV to RV pacing (23%), reduced effective treatment separation and likely attenuated the observed effect. Given its large sample size and weight in our analysis, BioPace likely moderated the pooled estimates, contributing to the attenuated overall effect observed [26]. Whether this pattern reflects inherent population characteristics or effect modification by systolic function will be explored in subsequent subgroup and meta-regression analyses.

The PACE trial showed that BiV pacing prevented adverse cardiac remodeling and preserved systolic function, while RV pacing led to deterioration of the LV volume and function [25]. The PREVENT-HF trial reported no significant difference between the two groups [27]. A RCT also reported that BiV pacing preserves LV function and dyssynchrony in patients with high-grade AV block [15]. Systematic reviews and meta-analyses confirm that BiV pacing better preserves or slightly improves LVEF and reduces adverse remodeling compared to RV pacing, but data on significant clinical outcomes such as mortality is limited [40, 41].

Subgroup analyses stratified by LVEF (< 50% vs. ≥ 50%) showed no significant interaction, indicating that baseline LVEF did not modify the effect of BiV pacing. The point estimates consistently favored BiV pacing for both mortality and HF hospitalization, contrasting with Lu et al.’s findings of greater benefit in reduced LVEF subgroups [14]. For HF hospitalization, the overall effect remained significant, though subgroup analyses were likely underpowered due to smaller samples and exclusion of BioPace [26]. For 6MWD, no significant interaction was observed; a marginal trend favoring RV pacing in the reduced LVEF subgroup likely reflects transient hemodynamic effects of RV apical pacing in compromised ventricles rather than a true treatment difference [42, 43]. The significant interaction between VP burden and HF hospitalization instead suggests greater BiV benefit with increasing pacing burden, a pattern physiologically plausible, as prolonged RV pacing can induce dyssynchrony and adverse remodeling, whereas BiV pacing maintains synchronized activation and prevents these effects [15, 40, 41]. Given that only two studies were included in the permanent VP subgroup, this observation warrants cautious interpretation.

The moderate heterogeneity observed in LVEF (%) at follow-up likely reflects variation in study design and population characteristics. Follow-up periods ranged from 3 months per arm in the crossover HOBIPACE to 4.8 ± 1.5 years in PACE, potentially influencing the outcome [10, 25]. Half of the included studies evaluated populations with preserved LVEF and standard pacing indications [15, 16, 25, 27], whereas the remainder enrolled patients with heart failure or reduced LVEF [10, 13, 18, 28]. Among the preserved-EF studies, PACE was distinctive in including patients with normal baseline LVEF and long-term follow-up, where BiV pacing helped maintain ventricular function by preventing the gradual decline in LVEF, as observed in RV pacing. Given its duration and statistical weight, PACE accounted for most of the observed heterogeneity [25].

To further explore the influence of LVEF as a variable, we conducted meta-regression to assess whether baseline LVEF influenced mortality or HF hospitalization. Meta-regression (as shown in online supplementary appendix, Supplementary Fig. S8) showed no relationship between baseline LVEF and effect size for HF hospitalization (*p* = 0.5315) or mortality (*p* = 0.1815), confirming that the magnitude of the benefit of BiV pacing did not significantly vary by baseline LVEF. This supports the conclusion that pacing burden, rather than baseline LVEF, is the key determinant of BiV pacing benefit in AV block [2, 44, 45].

### Clinical implications

The 2021 ESC guidelines recommend CRT for patients with LVEF < 40% or pacing-induced cardiomyopathy with LVEF < 35%, while its role in those with preserved LVEF or narrow QRS remains uncertain [26, 46]. In this meta-analysis, BiV pacing reduced HF hospitalizations and preserved LVEF compared with RV pacing, suggesting that high pacing burden may justify BiV pacing irrespective of baseline LVEF [14, 47]. Although the BioPace trial, which included a broader preserved-LVEF population with longer follow-up, reported neutral findings [26], the overall evidence supports a burden-based rationale for CRT consideration. However, this potential benefit must be weighed against the higher procedural complexity, longer implant times, and greater cost associated with CRT compared with conventional pacing [48–50]. Collectively, these considerations highlight that CRT use should remain guided by a balanced assessment of procedural risk, cost, and patient characteristics rather than a broad expansion of current indications.

### Study limitations

This meta-analysis was limited by study-level data, preventing patient-level or adjusted subgroup analyses. Most trials enrolled older adults, where competing non-cardiac mortality may have attenuated observed effects. Although most studies focused on advanced AV block, BLOCK-HF did not clearly distinguish AV block severity [2], and BioPace enrolled a mixed population without stratifying outcomes by pacing indication [26]. This heterogeneity may have diluted treatment effects. Additionally, variations in endpoints and follow-up durations, and RV lead positions may have further influenced outcomes, as most studies used apical RV pacing, which causes greater dyssynchrony and may exaggerate BiV benefit; septal pacing preserves synchrony and could narrow this gap [51, 52]. No cost-effectiveness analyses were reported across studies. BioPace data could not be included in the HF hospitalization subgroup analysis due to unavailability [26]. Lastly, while 6MWD did not show significant differences, this outcome may gain importance with data from future trials.

Further studies should confirm the benefits of burden-driven CRT in diverse pacing-dependent populations, particularly those with preserved LVEF, to strengthen the evidence base for guideline revision.

## Conclusion

In this meta-analysis of 3,685 patients with AV block and high VP burden, BiV pacing reduced HF hospitalizations by 17% and better preserved systolic function compared with RV pacing. Mortality differences were not significant. The consistent burden-dependent effect suggests that pacing burden may be an important determinant of BiV benefit, irrespective of the baseline LVEF. While these findings support consideration of a burden-based pacing approach, CRT use should remain guided by careful assessment of patient characteristics, procedural risk, and resource implications.

## Supplementary Information

Supplementary Material 1

## Data Availability

Data generated or analyzed during this study are included in this published article and its supplementary information files.

## Abbreviations

AF: Atrial Fibrillation
AV: Atrioventricular
AVB: Atrioventricular Block
AVJ: Atrioventricular Junction
BiV: Biventricular
CI: Confidence Interval
CMA: Comprehensive Meta-Analysis
CRT: Cardiac Resynchronization Therapy
CV: Cardiovascular
HF: Heart Failure
LBBB: Left Bundle Branch Block
LV: Left Ventricle/Left Ventricular
LVEF: Left Ventricular Ejection Fraction
MD: Mean Difference
PRISMA: Preferred Reporting Items for Systematic Reviews and Meta-Analyses
PROSPERO: International Prospective Register of Systematic Reviews
RCT: Randomized Controlled Trial
RoB: Risk of Bias
RR: Risk Ratio
SRMA: Systematic Review and Meta-Analysis
VP: Ventricular Pacing
6MWD: 6-Minute Walk Distance
