# Correction: Comparative effects of biventricular and right ventricular pacing on clinical outcomes in atrioventricular block: a systematic review and meta-analysis

**DOI:** 10.1186/s12872-025-05441-w

**Published:** 2025-12-20

**Authors:** Ayan Khalid, Anas Rasool, Shaikh Muhammad Daniyal, Muhammad Ibrahim, Fauzaan Ahmed Siddiqui, Muhammad Saad, Muhammad Hasan Siddiqui, Rahul Balach, Hafiz Bilal Ahmed, Isbah Gul, Yamaan Adil, Sabula Tabish, Danish Ali Ashraf, Ahmed Sajid, Romal Jabarkhil

**Affiliations:** 1 Department of Medicine, Dow University Of Health Sciences, Karachi, Pakistan; 2 Department of Medicine, Ziauddin University, Karachi, Pakistan; 3 Department of Medicine, TruGift Health LLC, Wilmington, DE USA; 4 Department of Medicine, Muzaffar Khan Mother and Children Hospital, Islamabad, Pakistan; 5 Department of Medicine, Ningarhar Regional Hospital, Jalalabad, Afghanistan

**Correction: BMC Cardiovascular Disorders 25, 835 (2025)**

**https://doi.org/10.1186/s12872-025-05336-w**

In this article [1], the author’s name ‘Romal Jabarkhil’ was incorrectly written as ‘Romal Jabakhil’.

The original article has been corrected.
