# Supplementary material for: Comparative effects of biventricular and right ventricular pacing on clinical outcomes in atrioventricular block: a systematic review and meta-analysis
Source: BMC Cardiovasc Disord. 2025 Nov 25;25:835. doi: 10.1186/s12872-025-05336-w (PMC12649102; doi:10.1186/s12872-025-05336-w)

**Supplementary online appendix**

**Online supplementary Table 1**

| Database or Search Engine | String | Results |
| --- | --- | --- |
| PubMed | \| **("biventricular pacing" OR "ventricular resynchronization" OR "cardiac resynchronization therapy" OR "BiV pacing")** \|  \|  \| \| --- \| --- \| --- \|  \| **AND** \|  \|  \| \| --- \| --- \| --- \|  \| **("atrioventricular block" OR "AV block" OR "atrioventricular junction ablation" OR "AVJ ablation")** \|  \|  \| \| --- \| --- \| --- \|  \|  \|  \|  \| \| --- \| --- \| --- \|  \| **AND** \|  \|  \| \| --- \| --- \| --- \|  \| **(mortality OR death OR "heart failure hospitalization" OR "HF hospitalization" OR "6-minute walk distance" OR 6MWD)** \| \| --- \| | 153 |
| Google Scholar | **allintitle: biventricular pacing AV block randomized trial mortality hospitalization 6MWD** | 270 |
| Cochrane Library | **(("biventricular pacing" OR "cardiac resynchronization therapy" OR "BiV pacing")  AND  ("atrioventricular block" OR "AV block" OR "atrioventricular junction ablation" OR "AVJ ablation")  AND  (mortality OR death OR "heart failure hospitalization" OR 6MWD OR "6‑minute walk"))** | 53 |
| ScienceDirect | **("biventricular pacing" OR "cardiac resynchronization therapy") AND ("atrioventricular block" OR "atrioventricular junction ablation") AND ("randomized controlled trial") AND ("all‑cause mortality" OR "hospitalization for heart failure" OR "six‑minute walk distance") AND ("follow‑up")** | 427 |

**Online Fig.S1a: Quality assessment with the Cochrane Risk of Bias In randomized Studies (Rob 2).
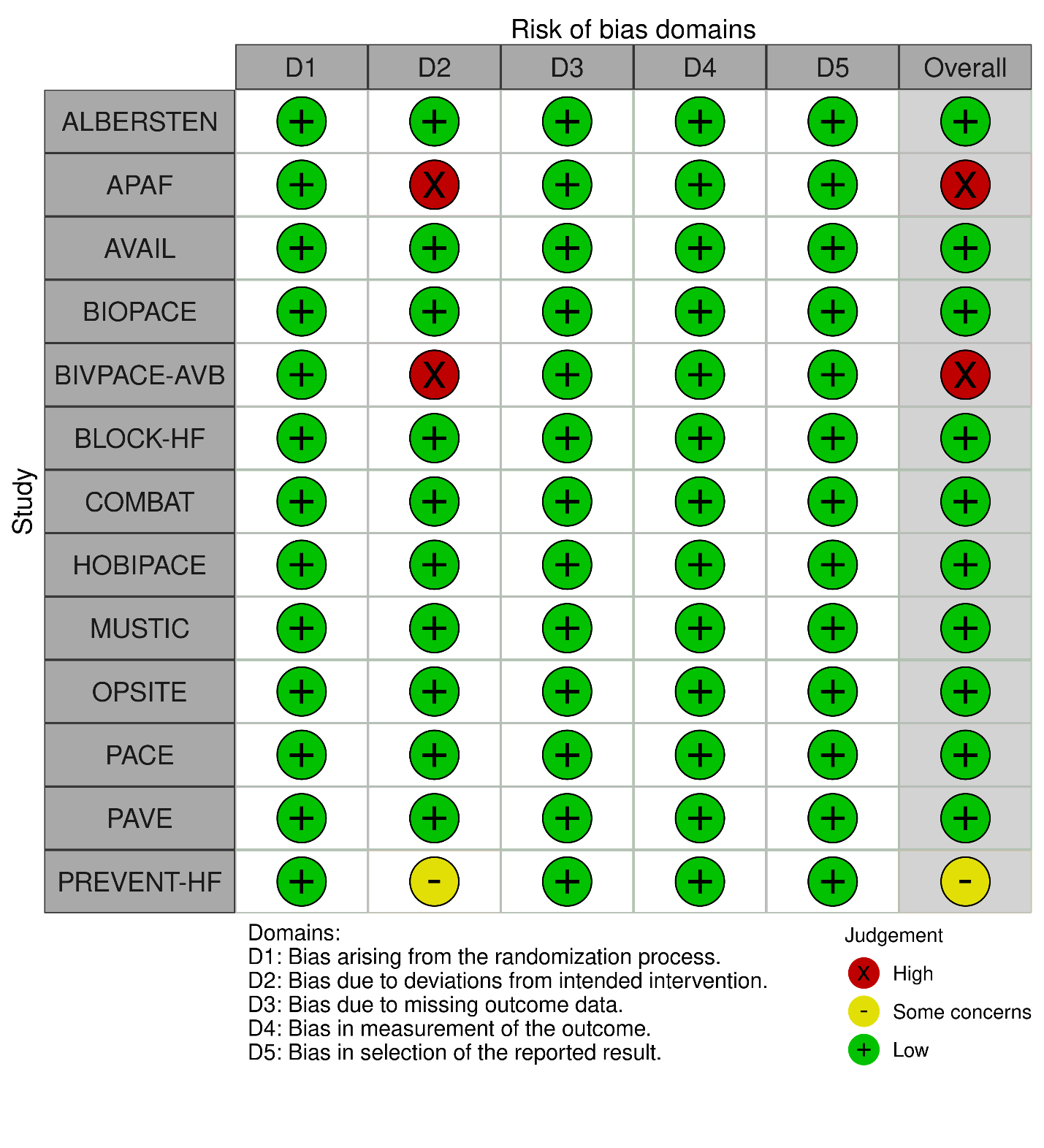
Online Fig.S1b: Quality assessment with the Cochrane tool for assessing risk of bias in randomised trials using weight distribution (RoB 2).
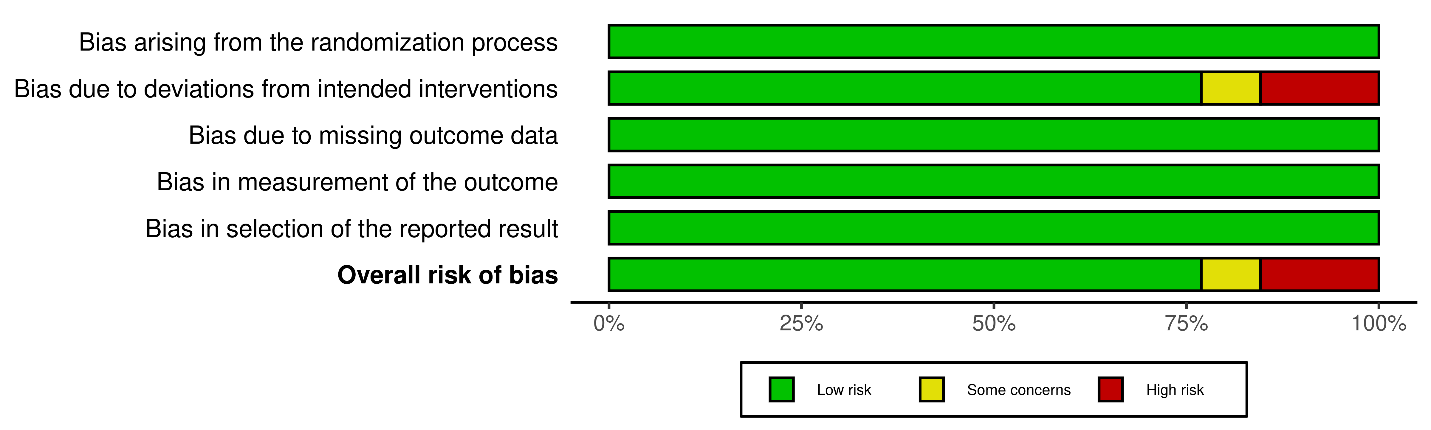
**

**Online Supplementary Fig.S2:** Subgroup analysis of (a) HF hospitalization, (b) Mortality, (c) 6-minute walk distance, (d) CV death and (e) LVEF (%) at follow-up based on baseline LVEF.
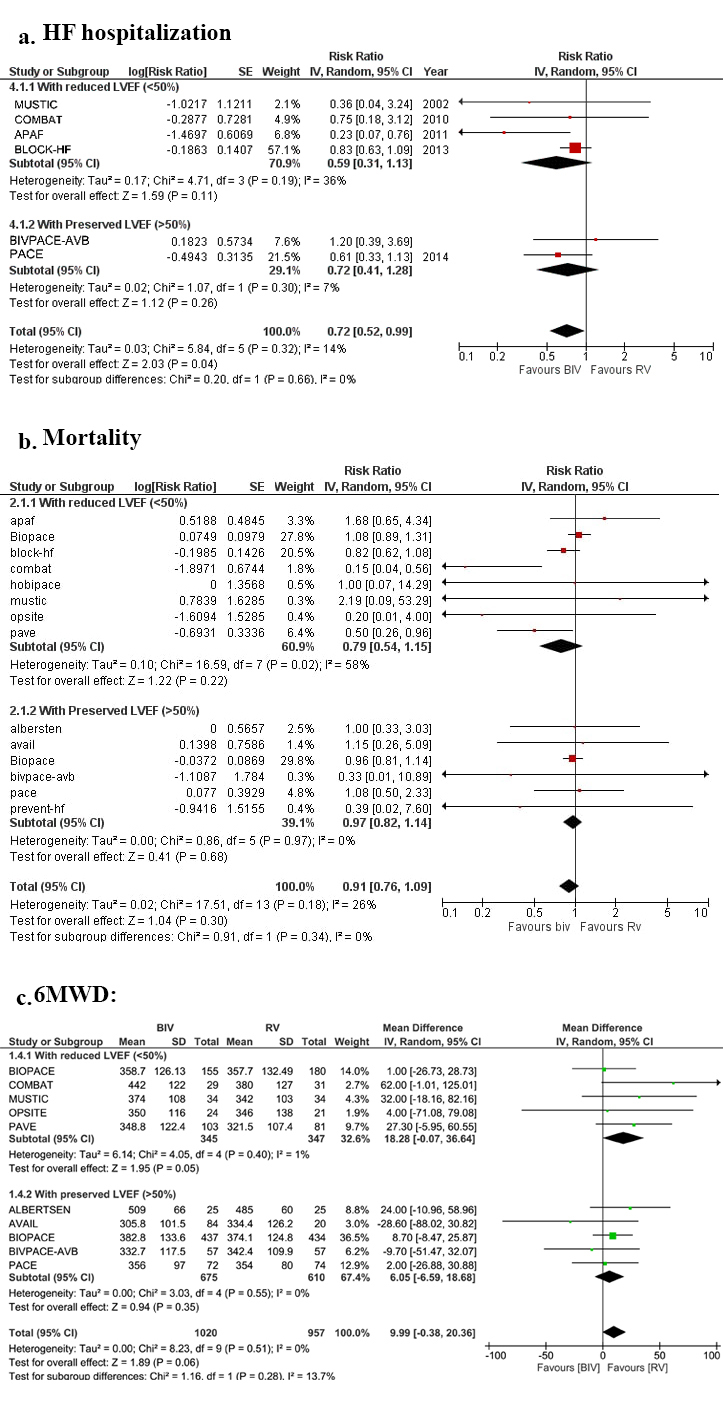


**Fig.S2d:**

**
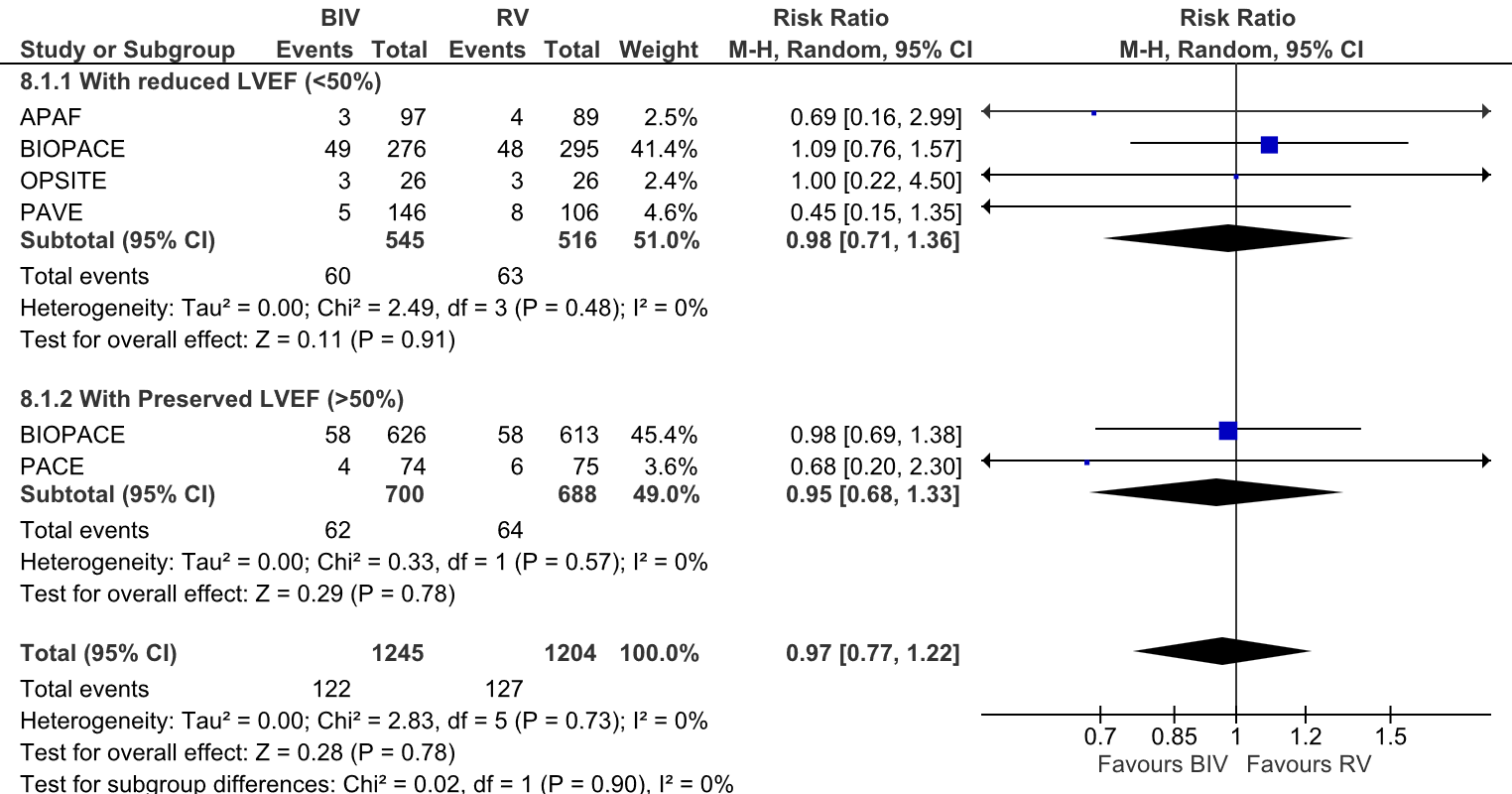
**

**Fig.S2e:**

**
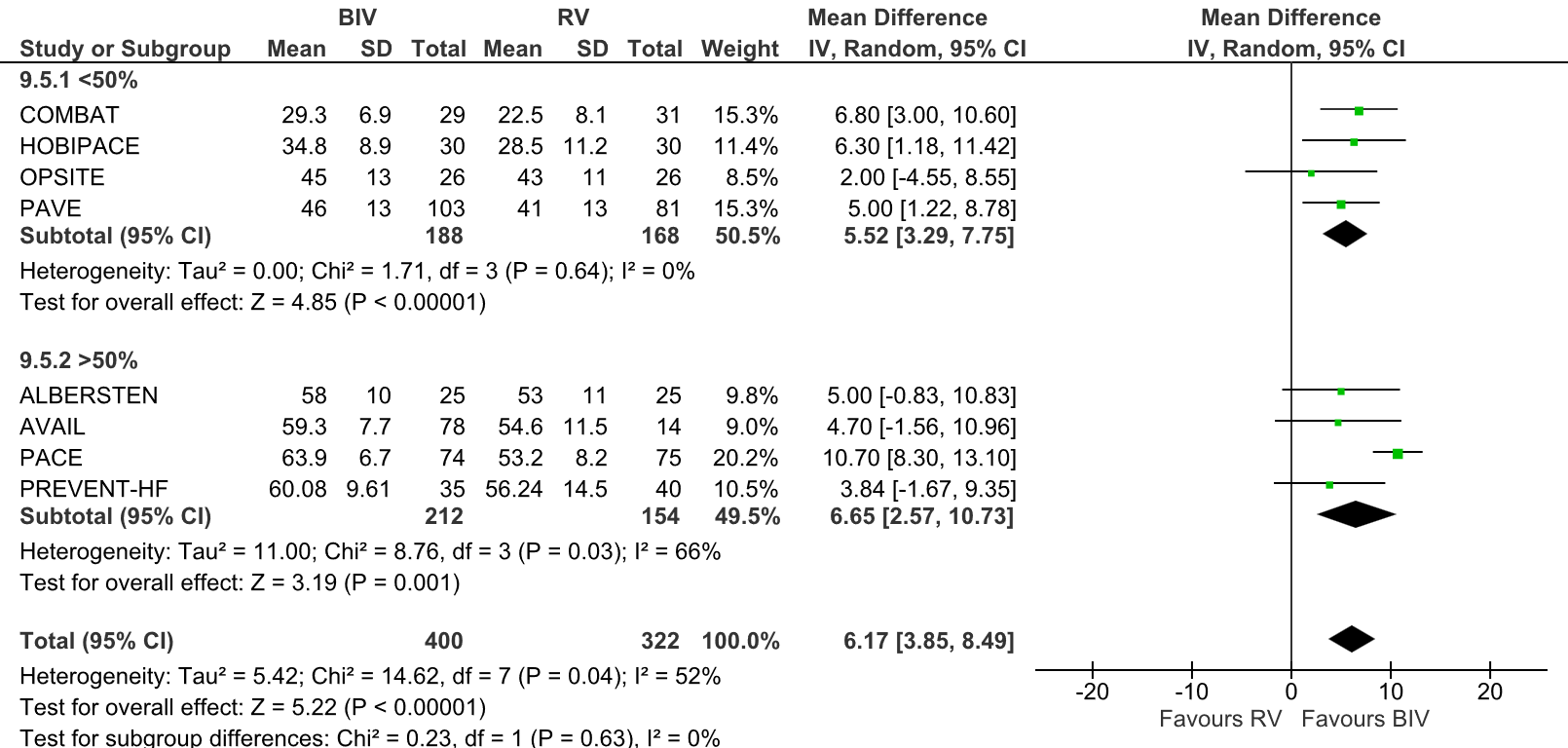
**

**Online Supplementary Fig.S3:** Subgroup analysis of (a) HF hospitalization, (b) Mortality, (c) 6-minute walk distance, and (d) LVEF (%) at follow-up based on RV pacing site.

**Fig.S3a:**


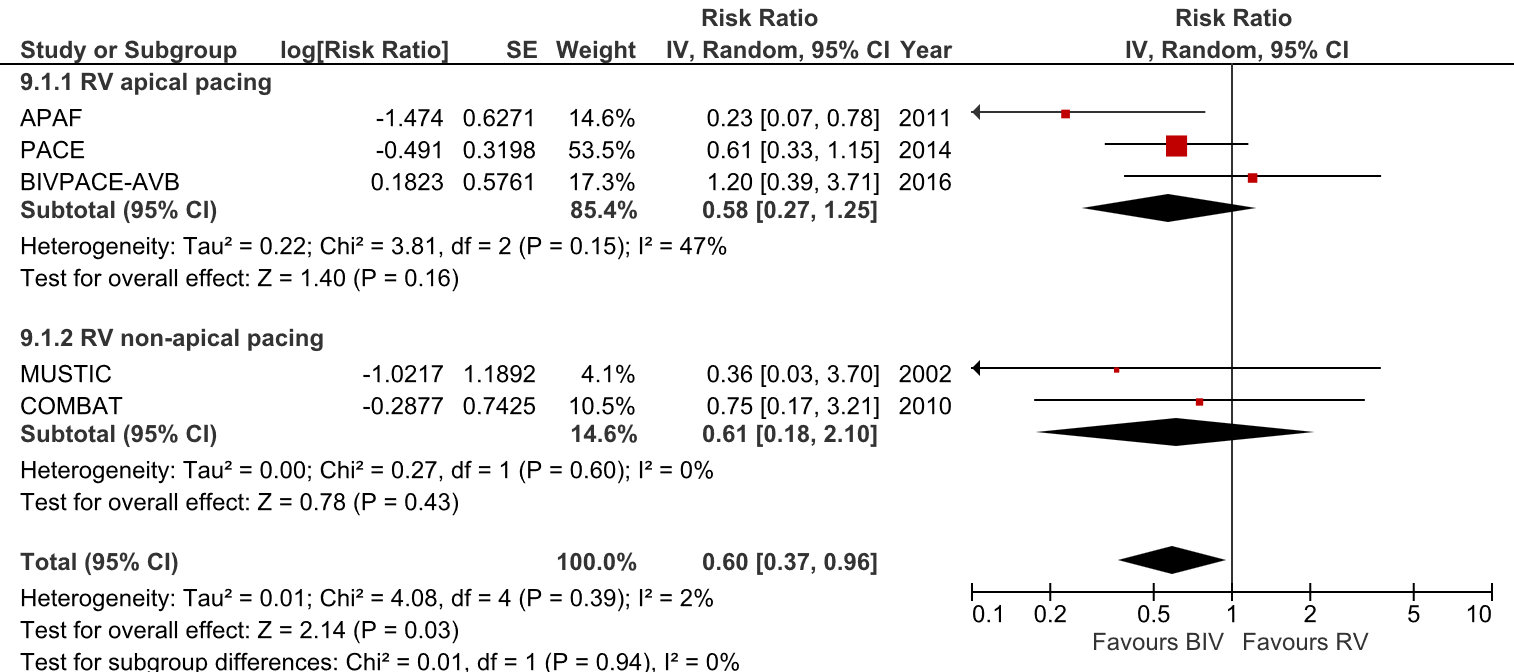


**Fig.S3b:**

**
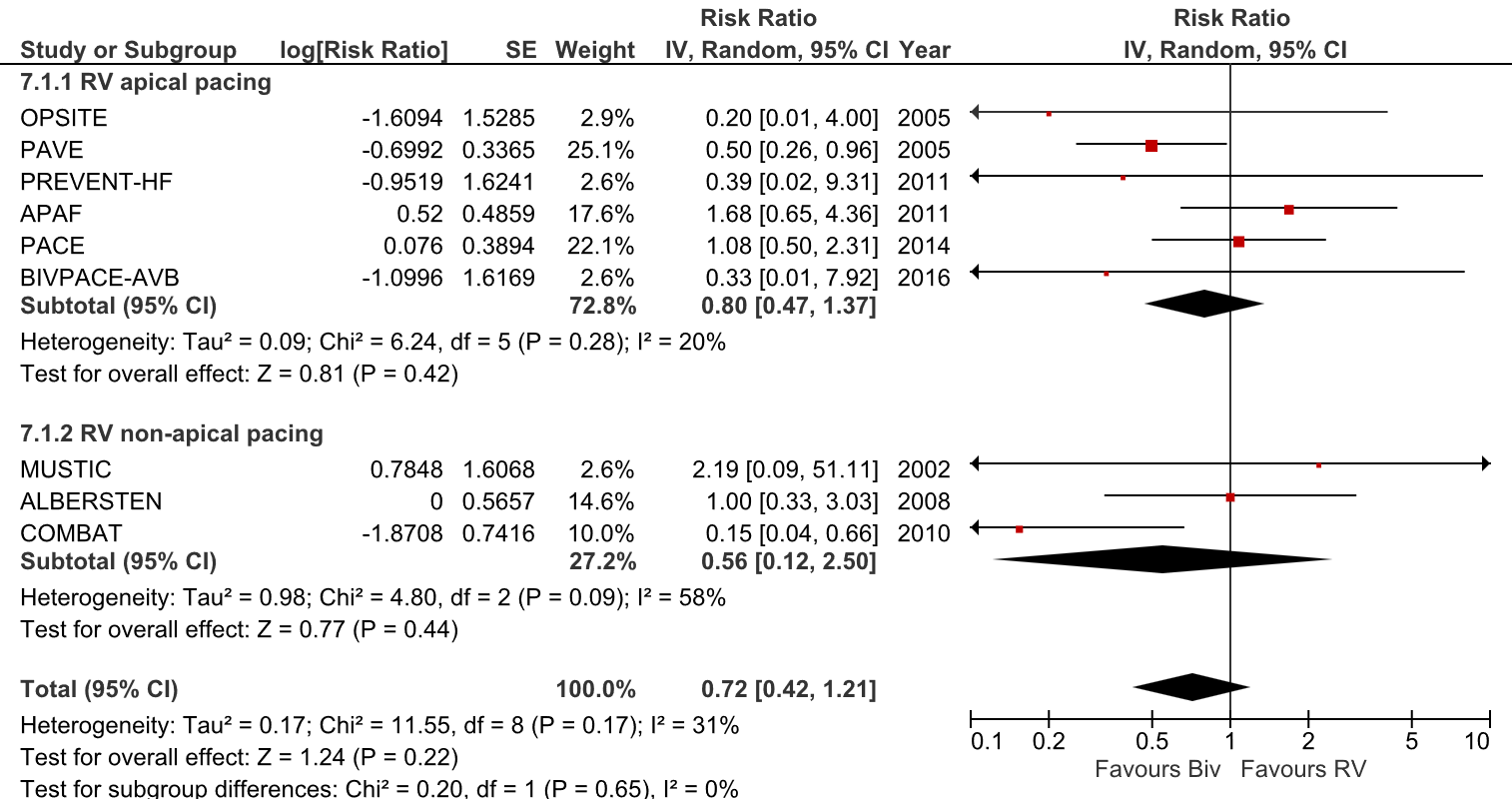
**

**Fig.S3c:**

**
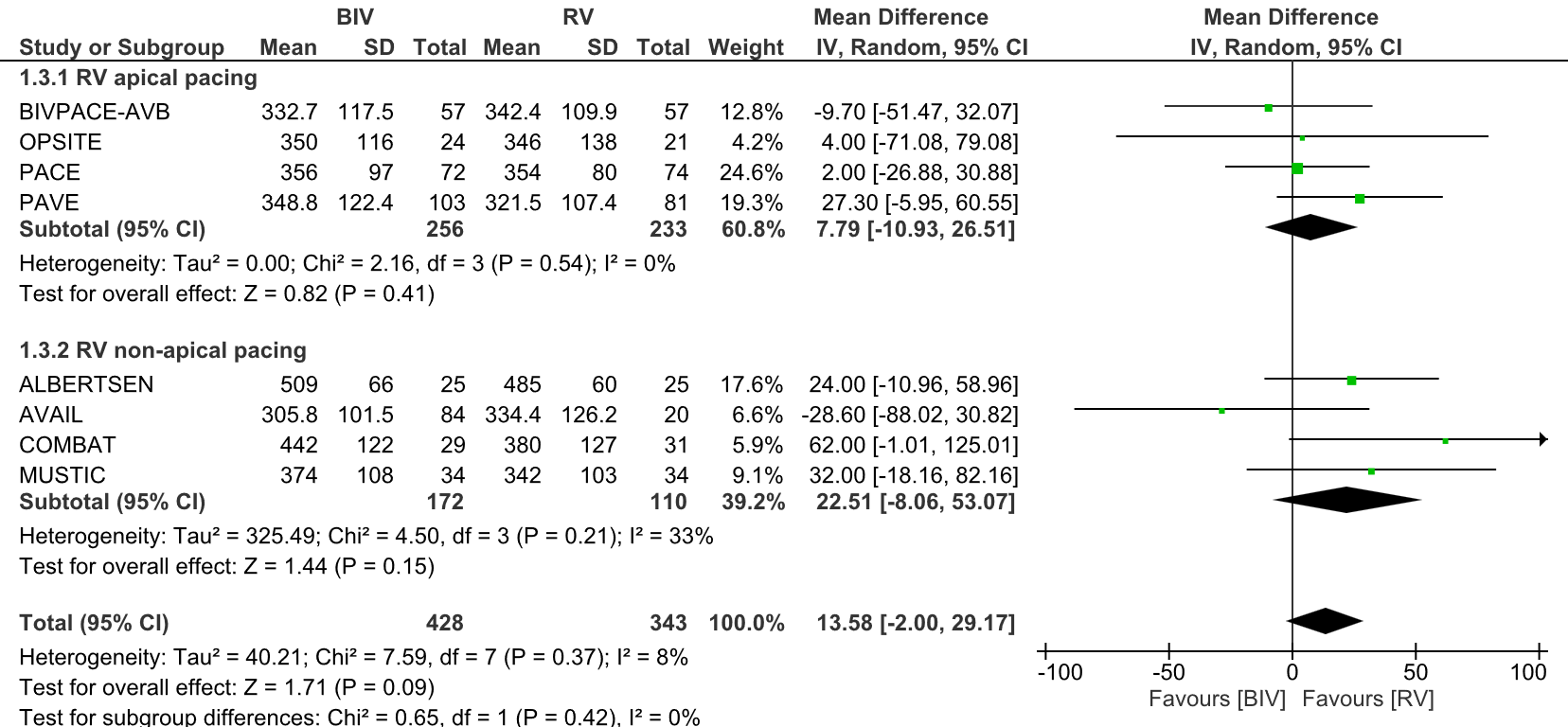
**

**Fig.S3d:**

**
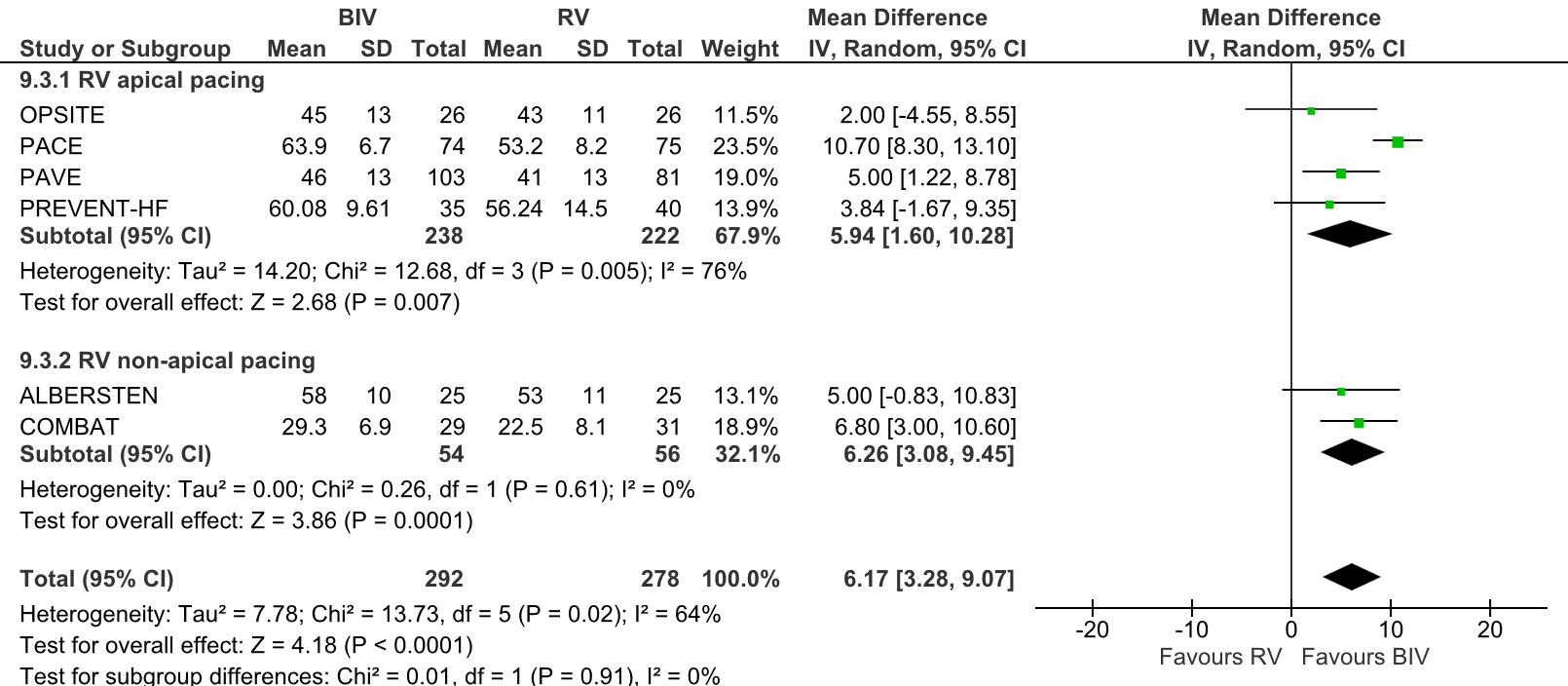
**

**Online Supplementary Fig.S4:** Subgroup analysis of (a) HF hospitalization, (b) Mortality, (c) 6-minute walk distance, (d) CV death, and (e) LVEF (%) at follow-up based on age.

**Fig.S4a:**

**
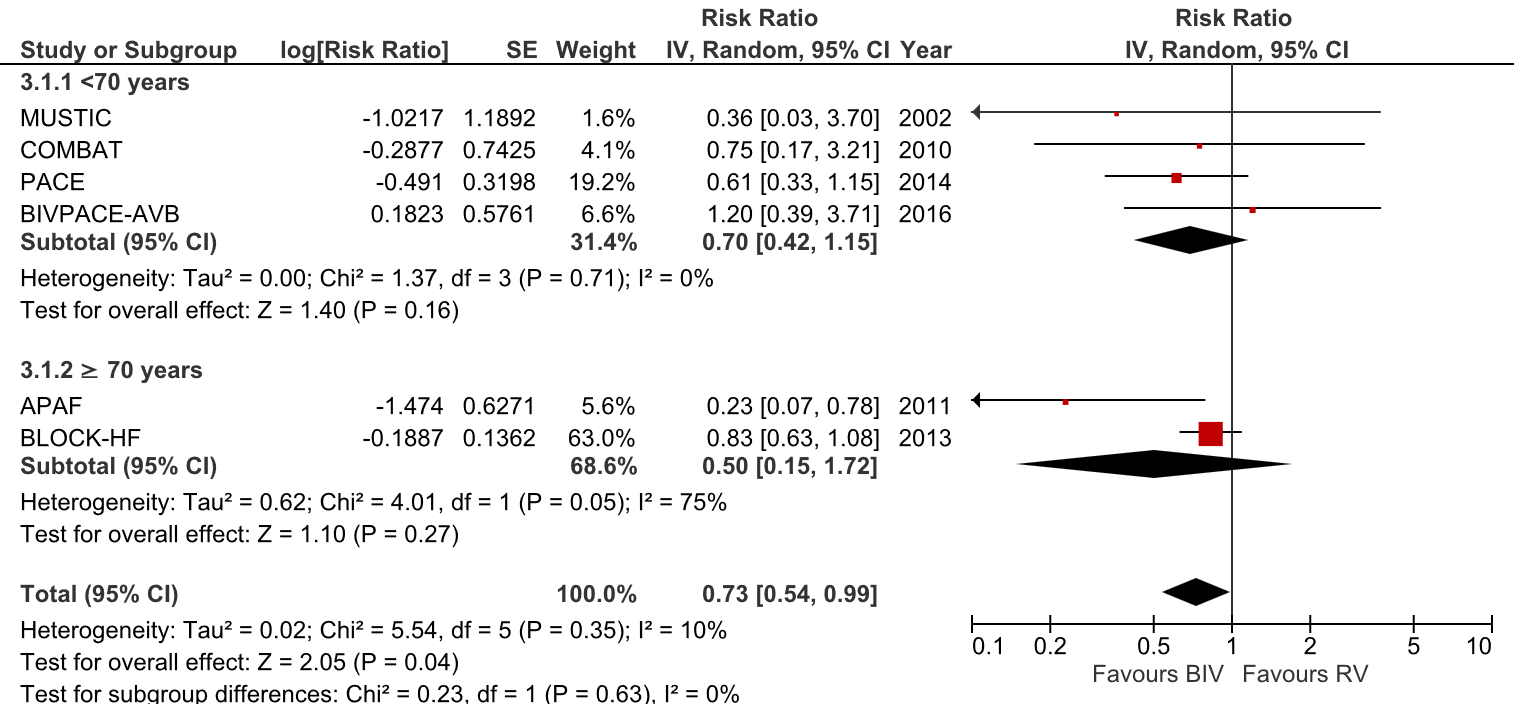
**

**Fig.S4b:**


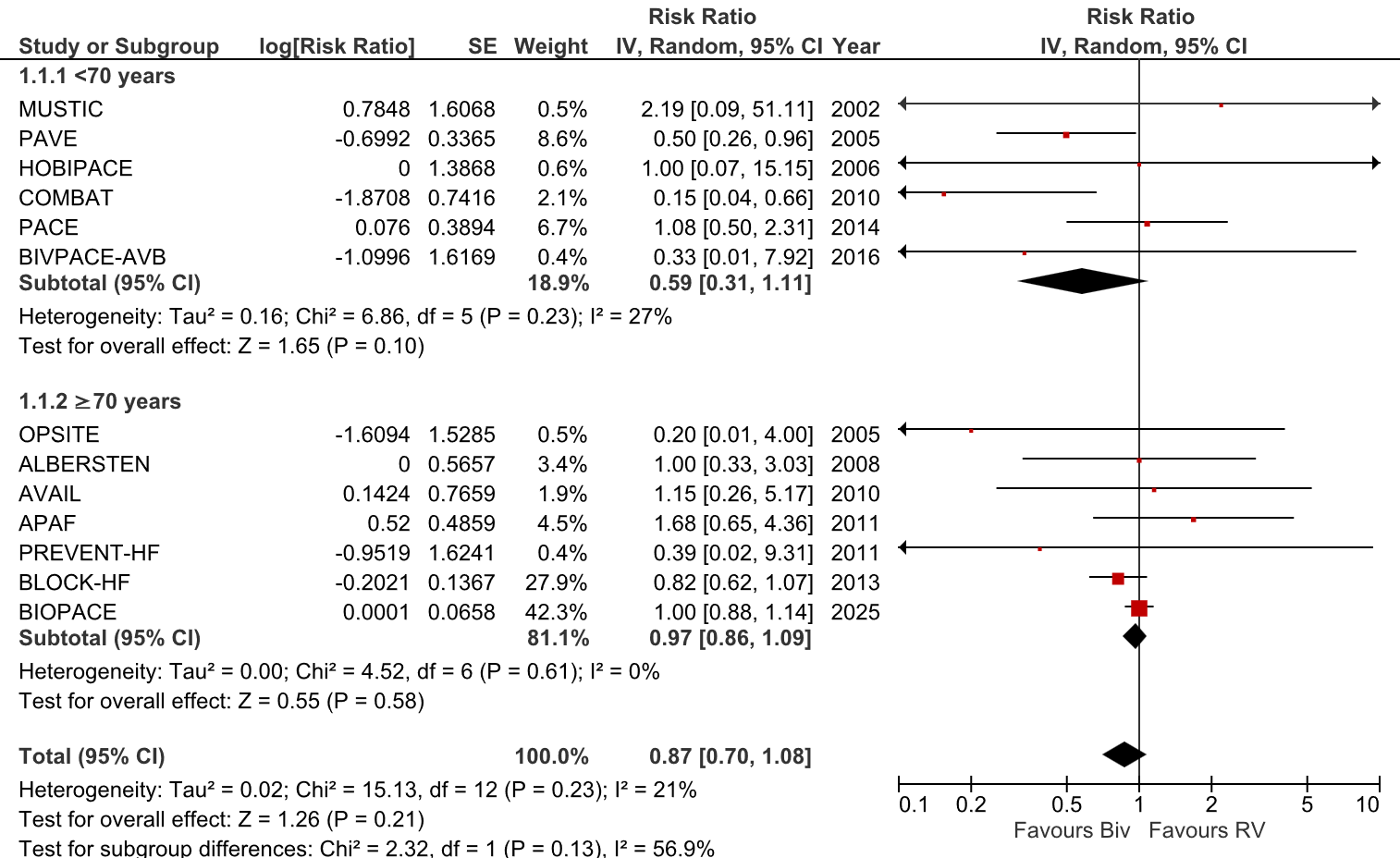


**Fig.S4c:**


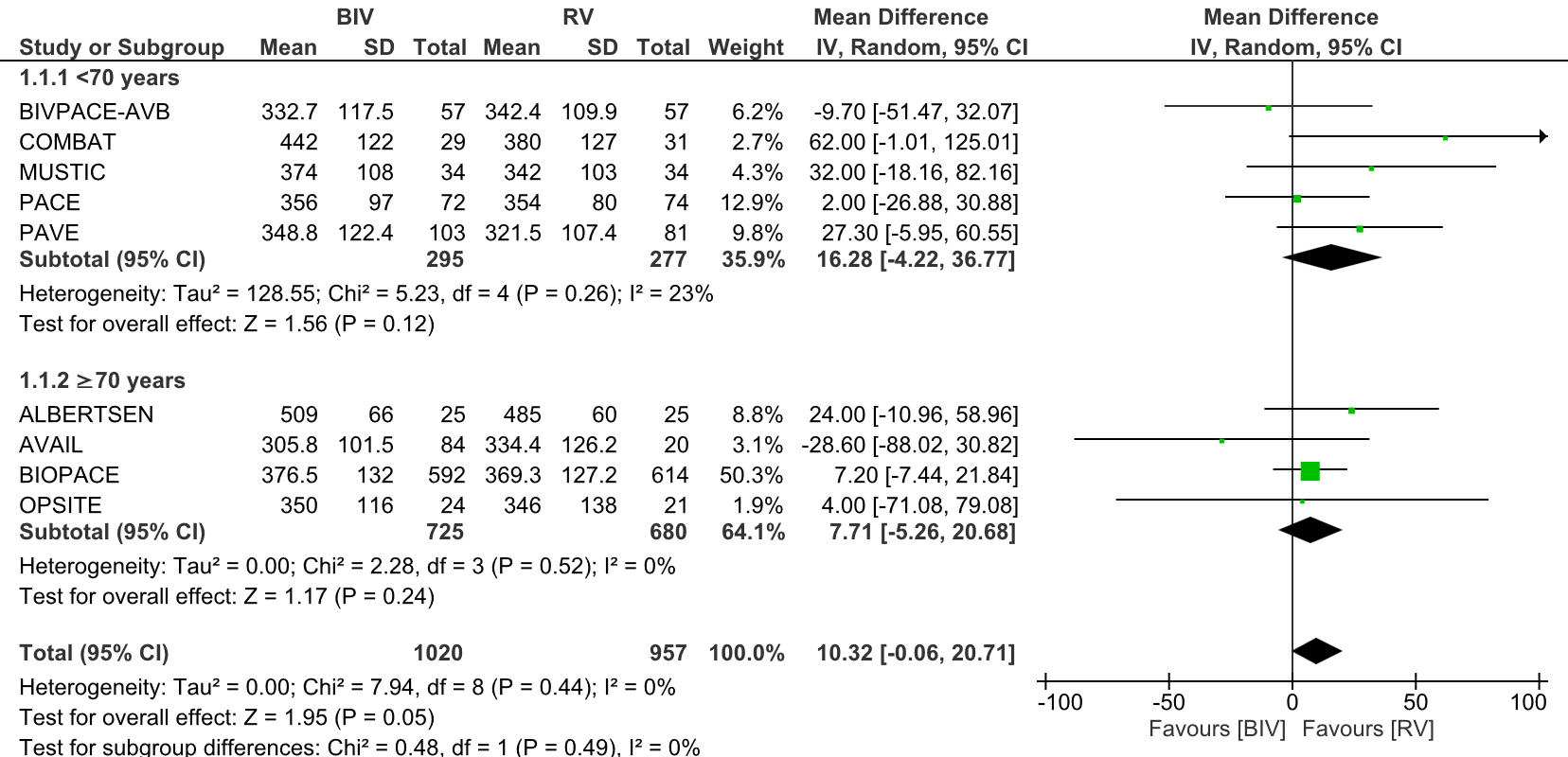


**Fig.S4d:**


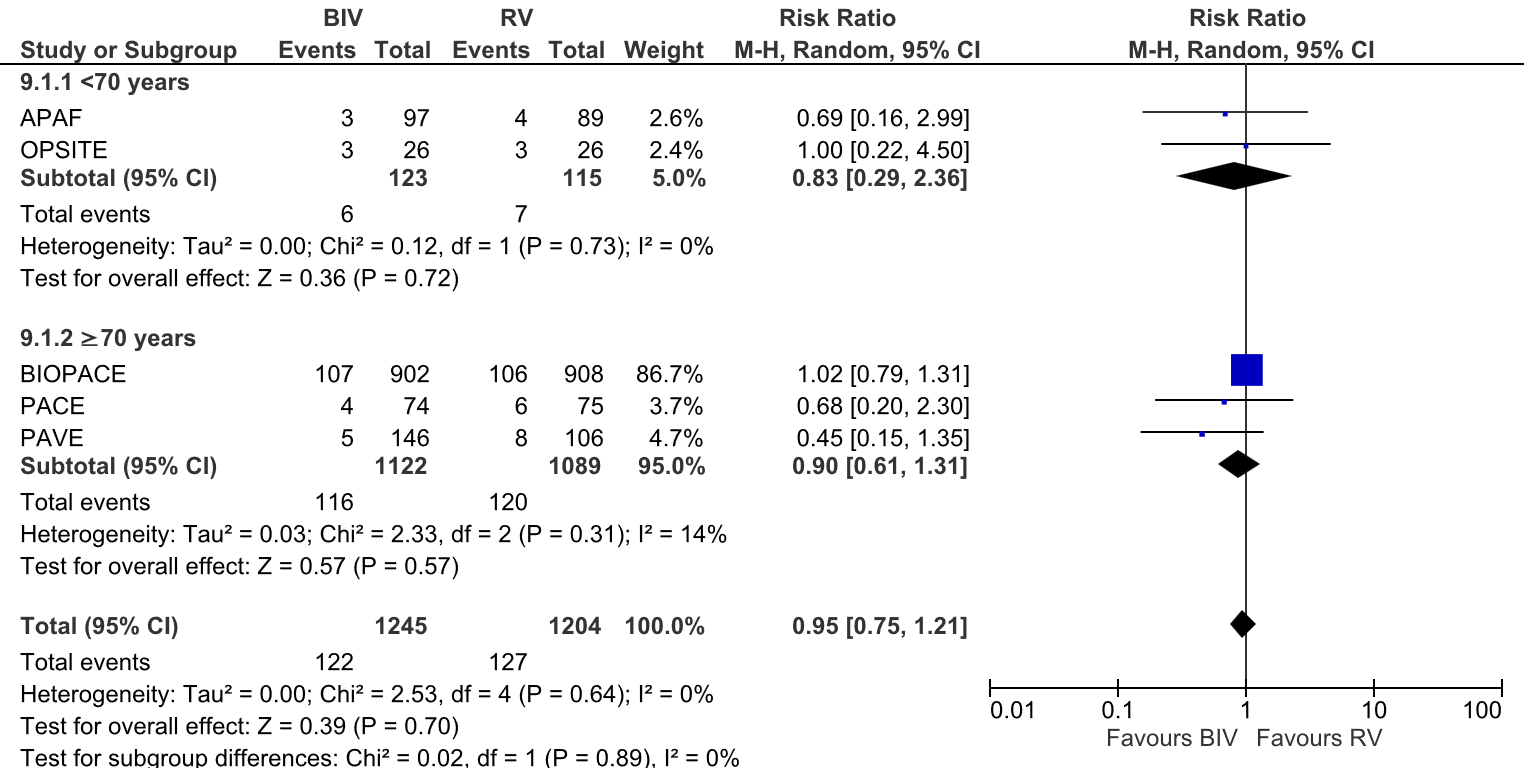


**Fig.S4e:**

**
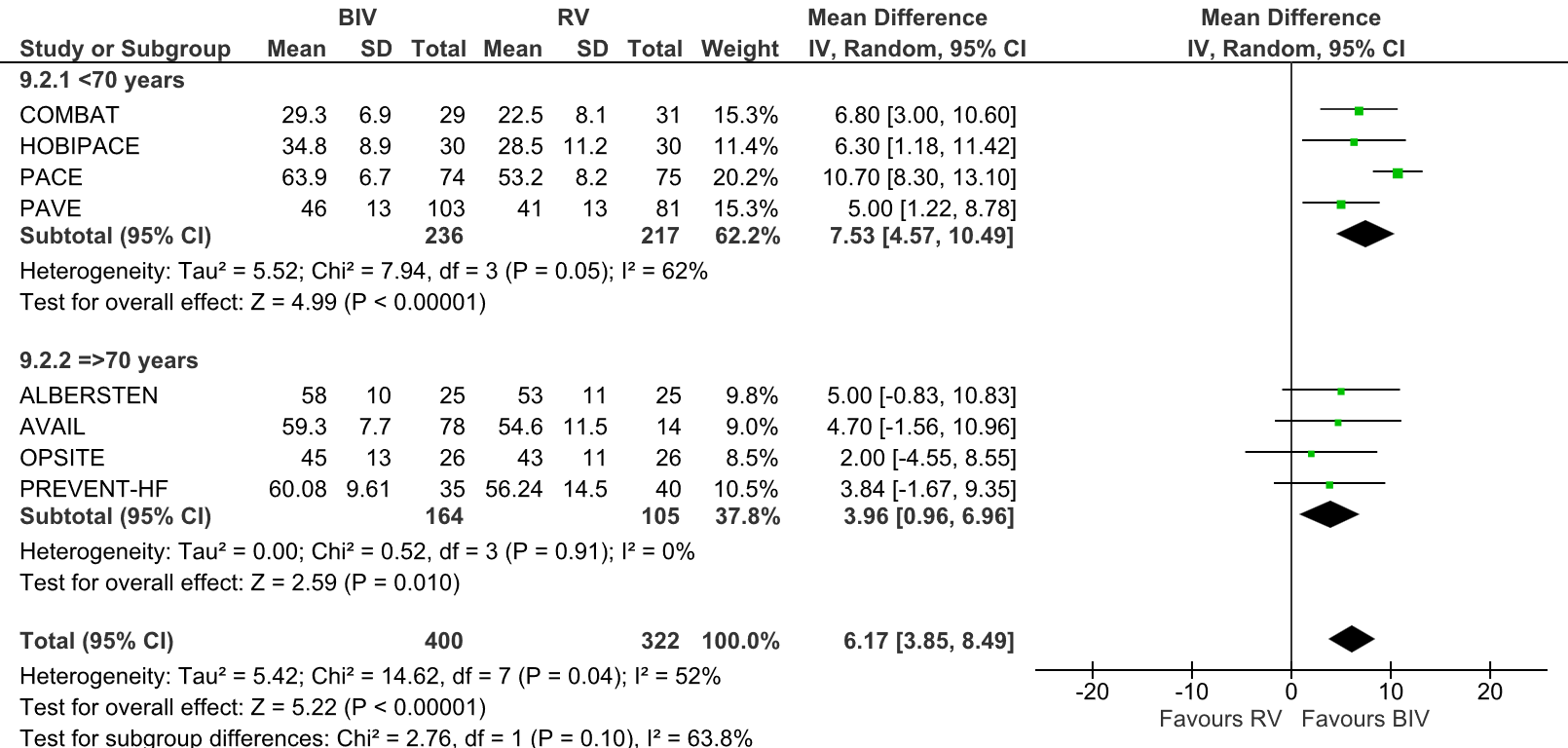
**

**Online Supplementary Fig.S5:** Subgroup analysis of (a) HF hospitalization, (b) Mortality, (c) 6-minute walk distance, and (d) LVEF (%) at follow-up based on VP burden.

**Fig.S5a:**


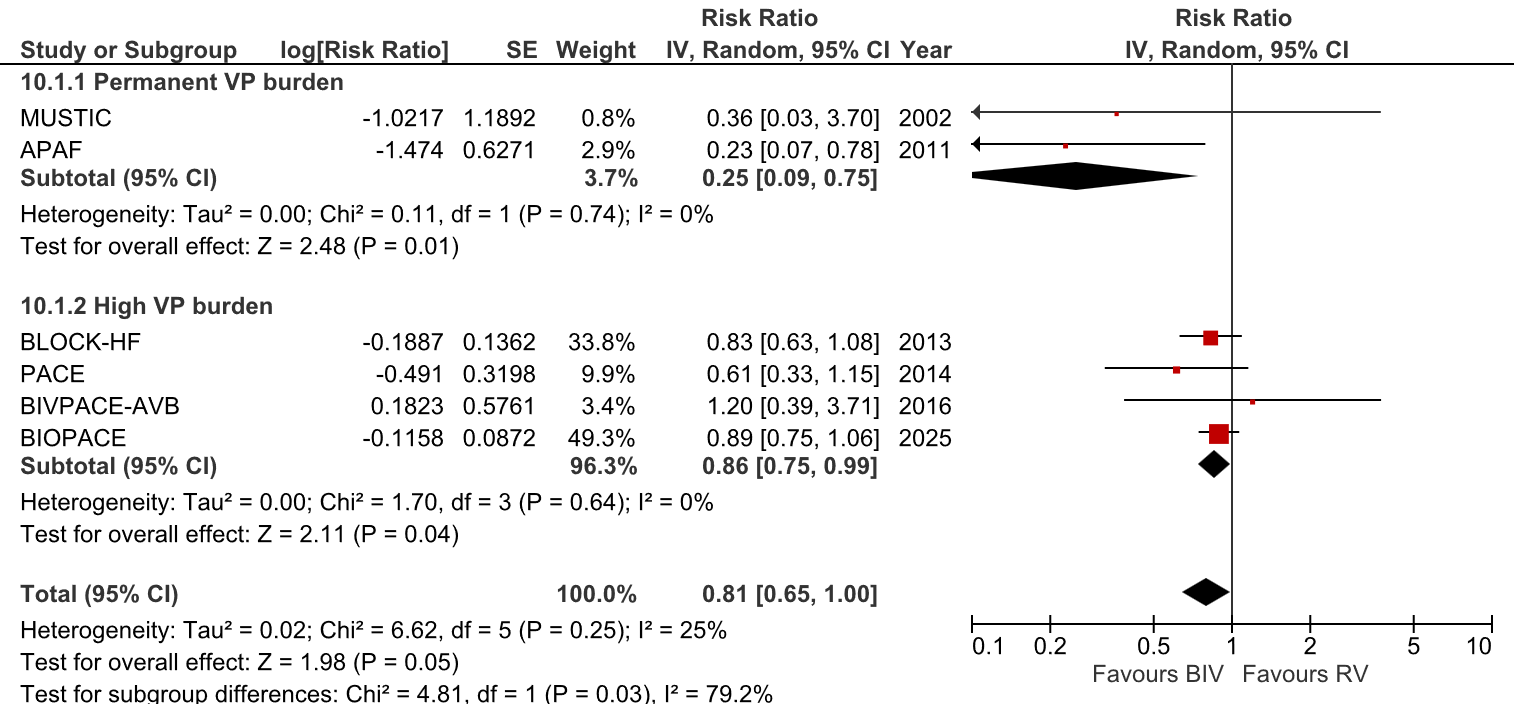


**Fig.S5b:**


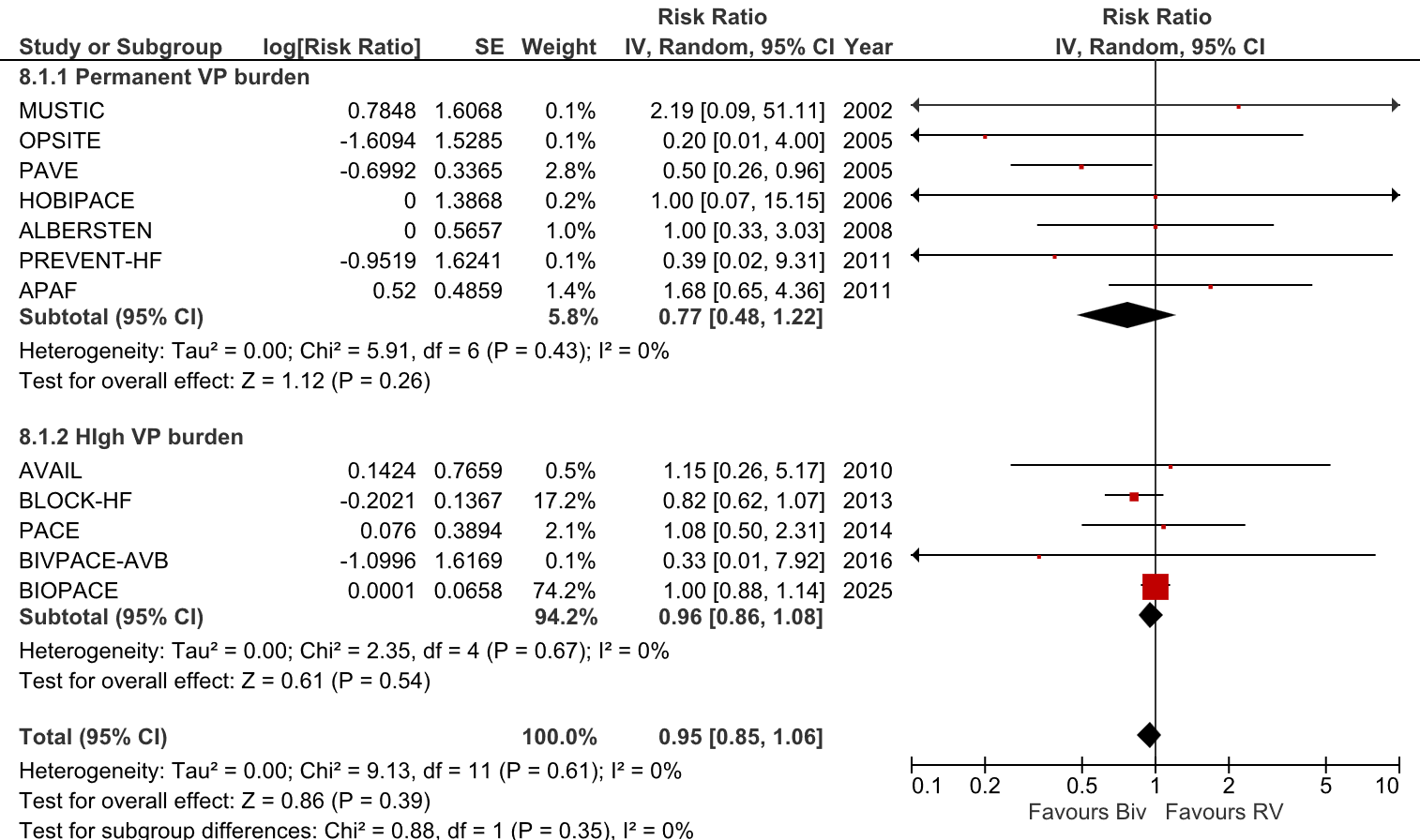


**Fig.S5c:**


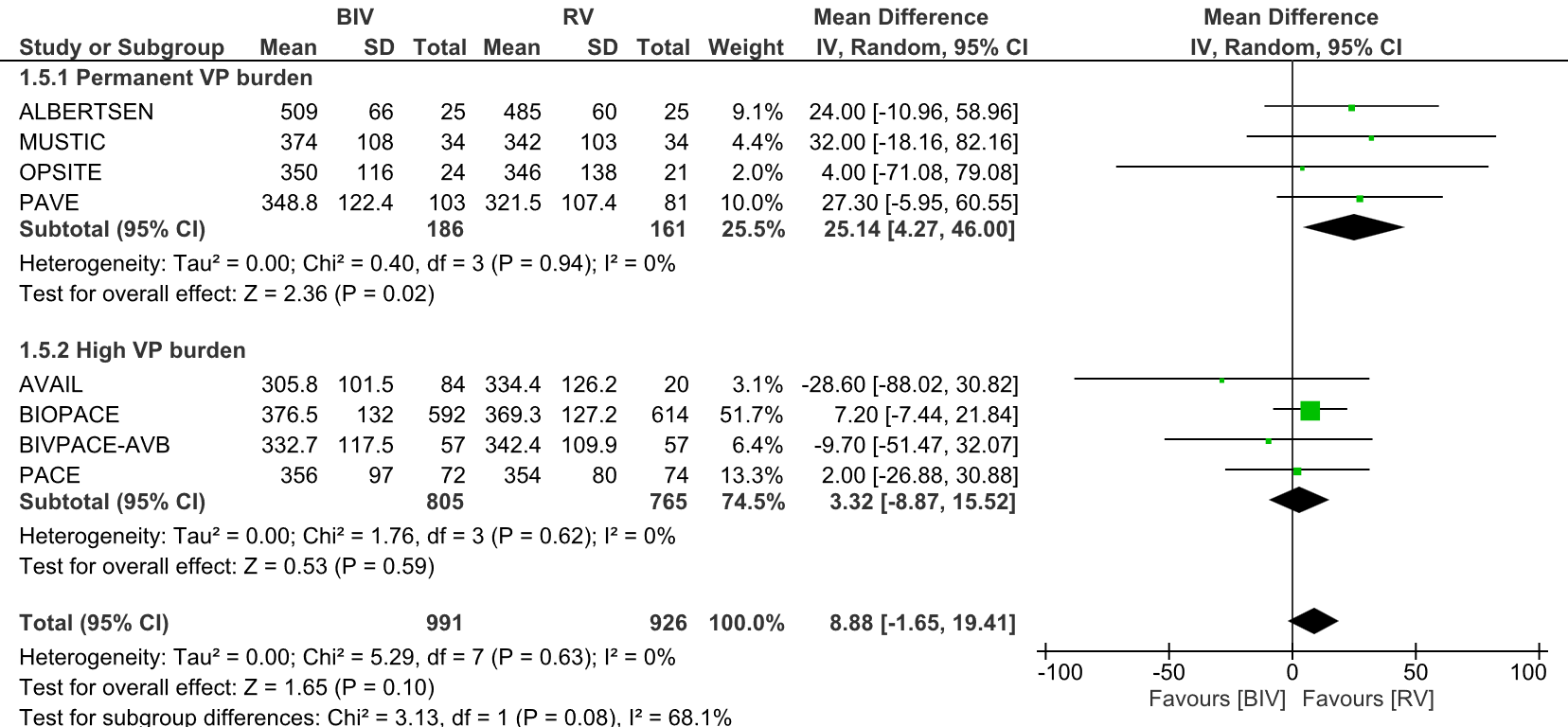


**Fig.S5d:**

**
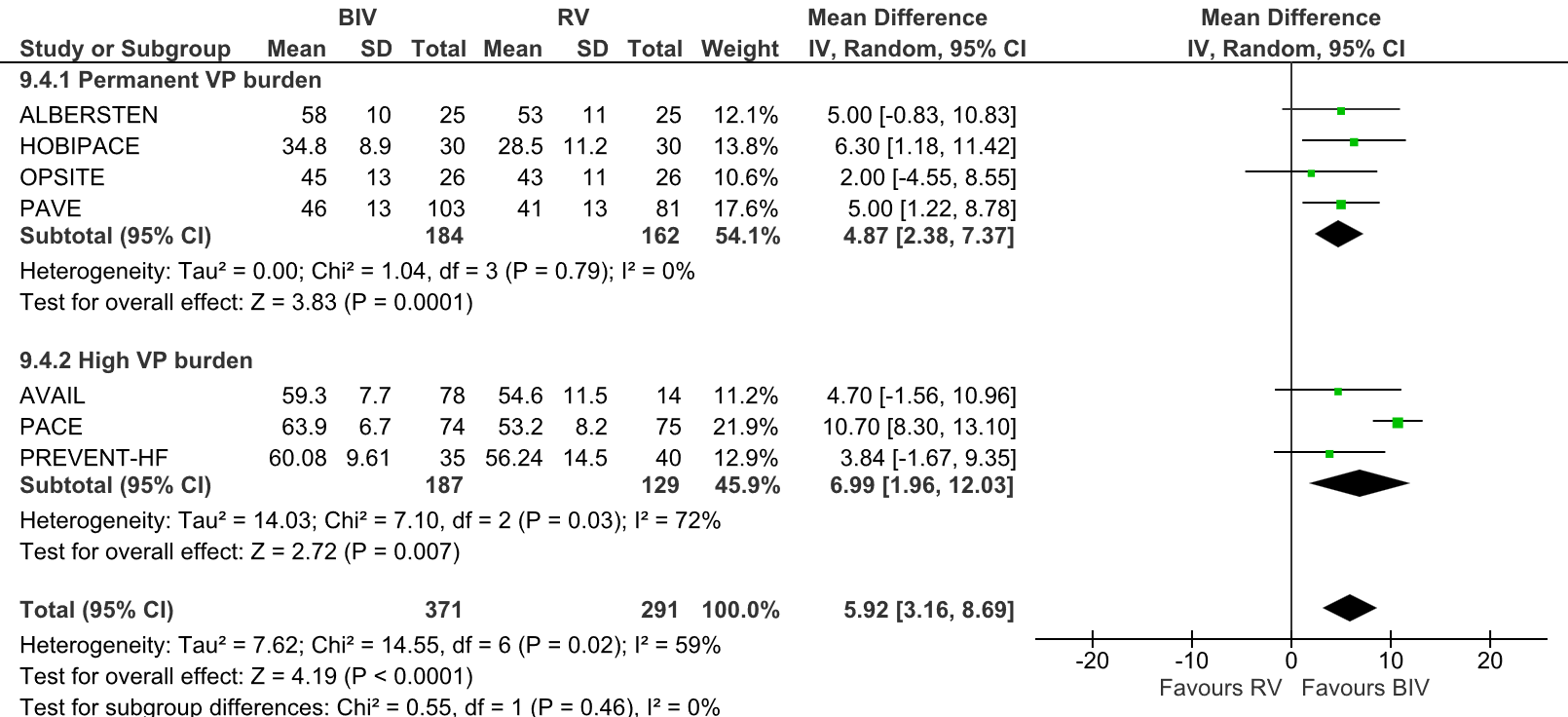
**

**Online Supplementary Fig.S6:** Sensitivity analysis of (a) HF hospitalization, (b) Mortality, (c) 6-minute walk distance, and (d) LVEF (%) at follow-up.


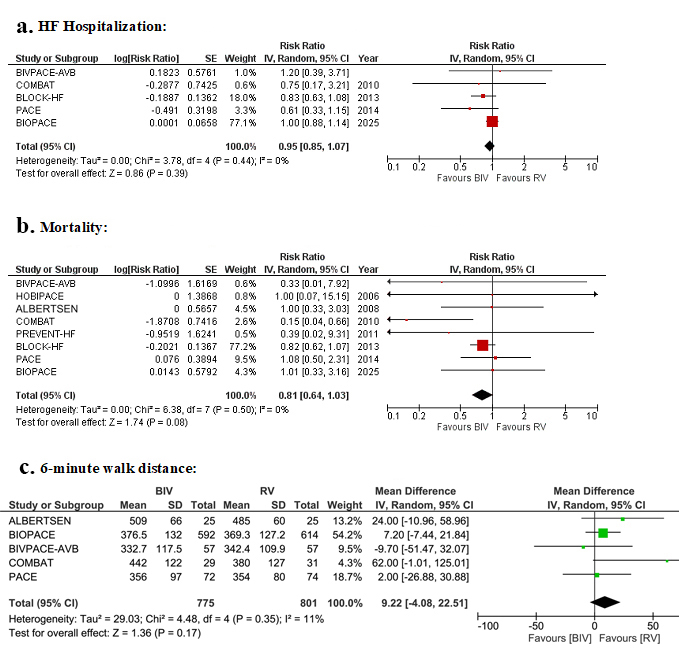


**Fig.S6d:**

**
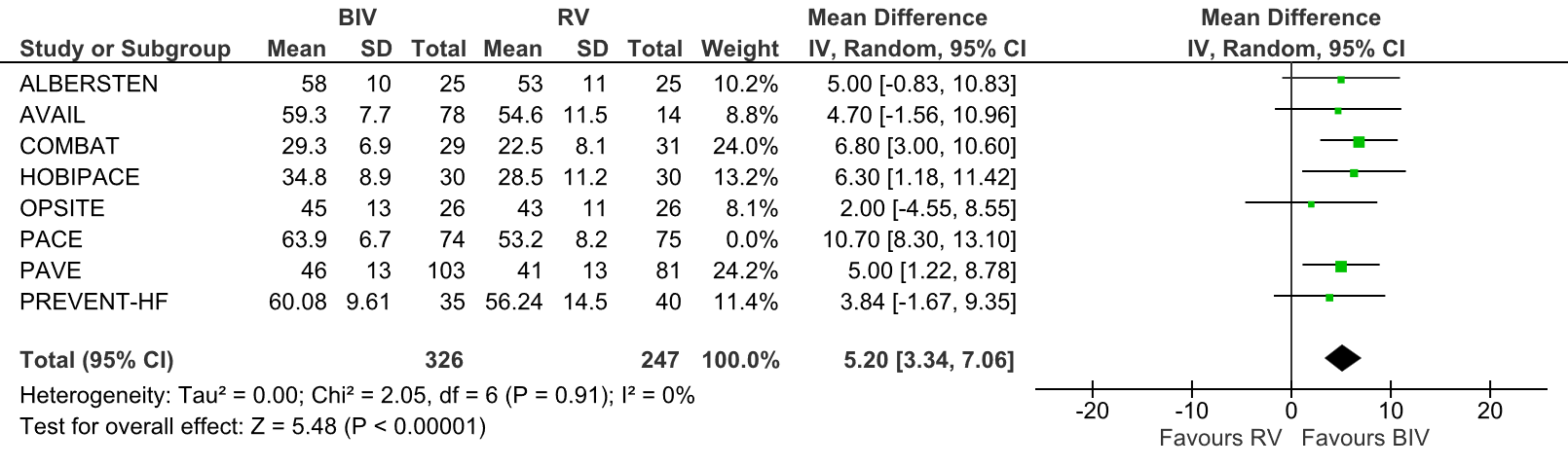
**

**Online supplementary Fig.S7:** Funnel plots

**Fig.S7a:** HF hospitalization


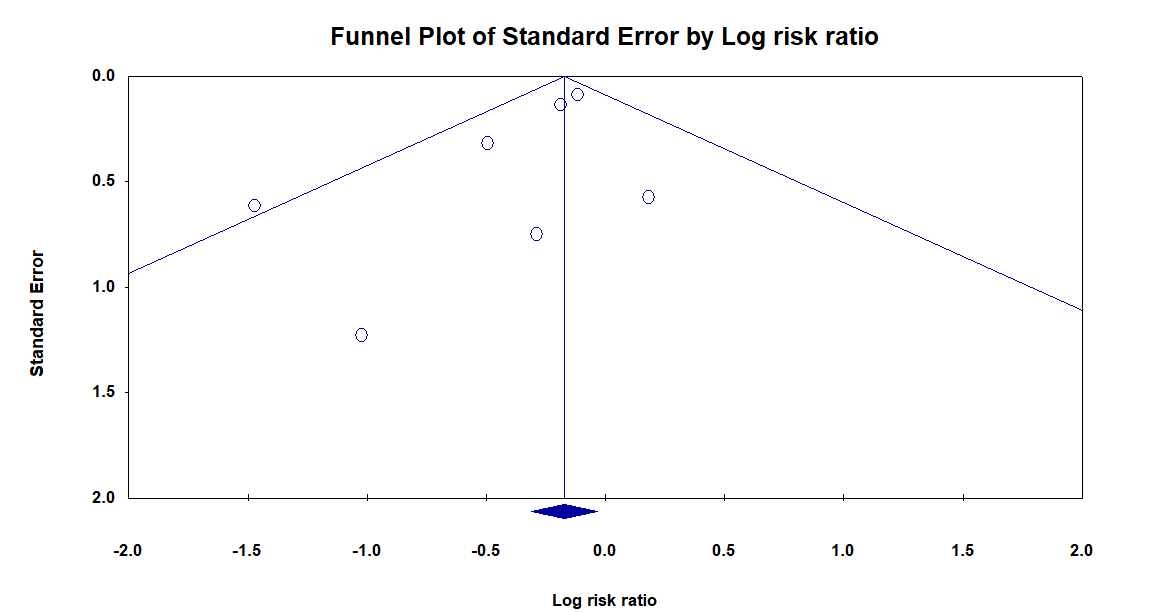


**Fig.S7b:** For Mortality


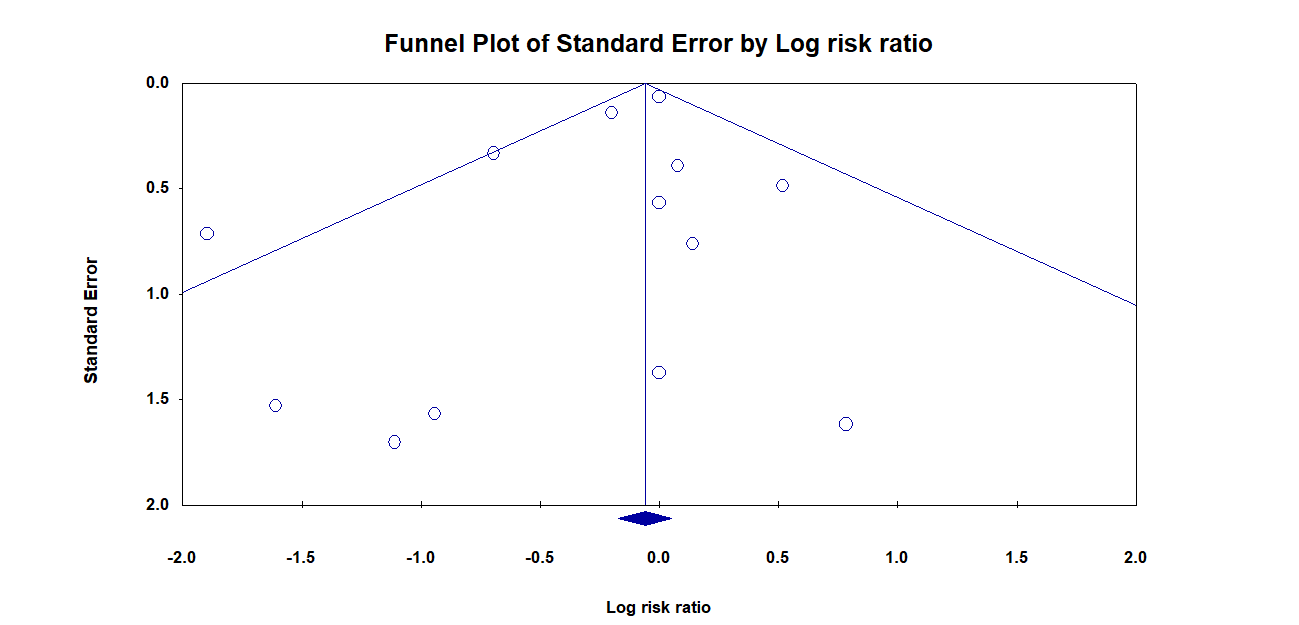


**Fig.S7c:** Six-minute walk distance


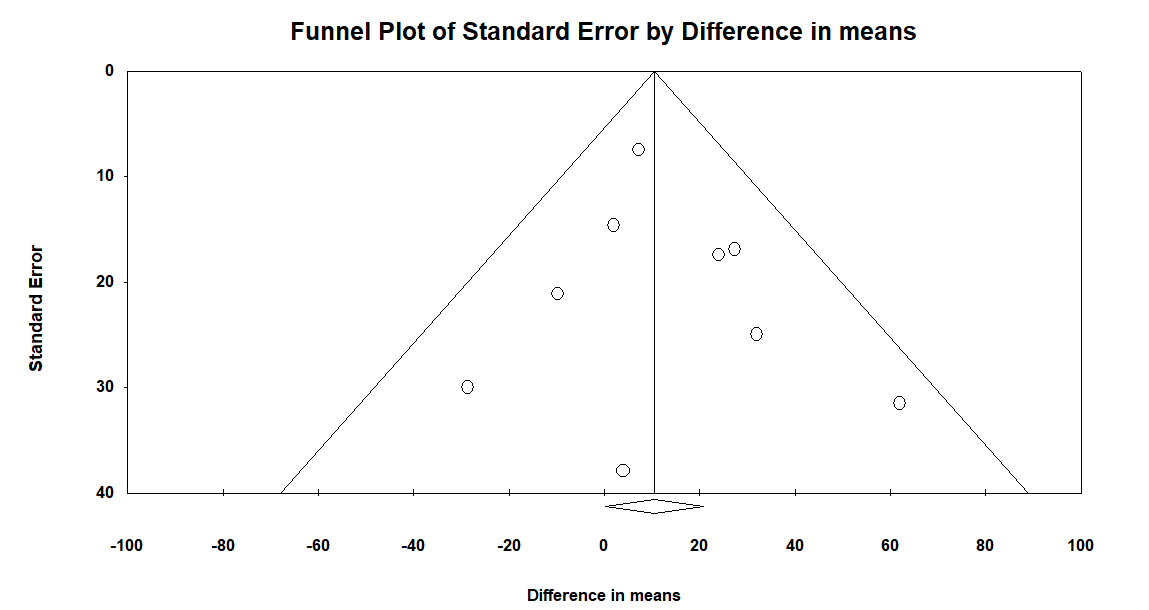


**Fig.S7d: CV death**

**
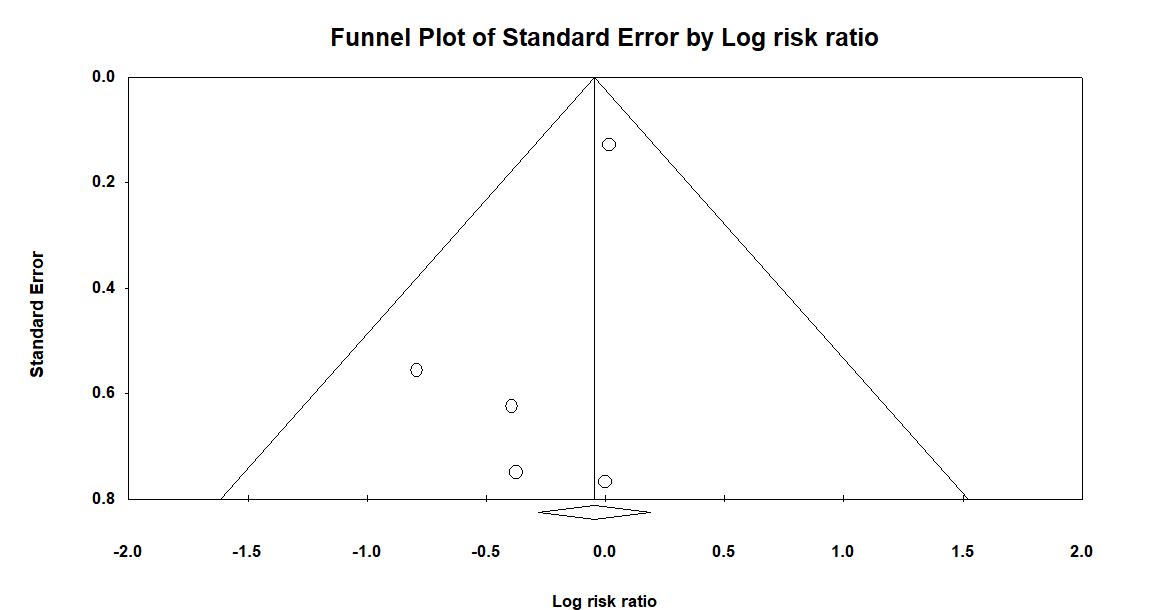
**

**Fig.S7e: LVEF (%) at follow-up**

**
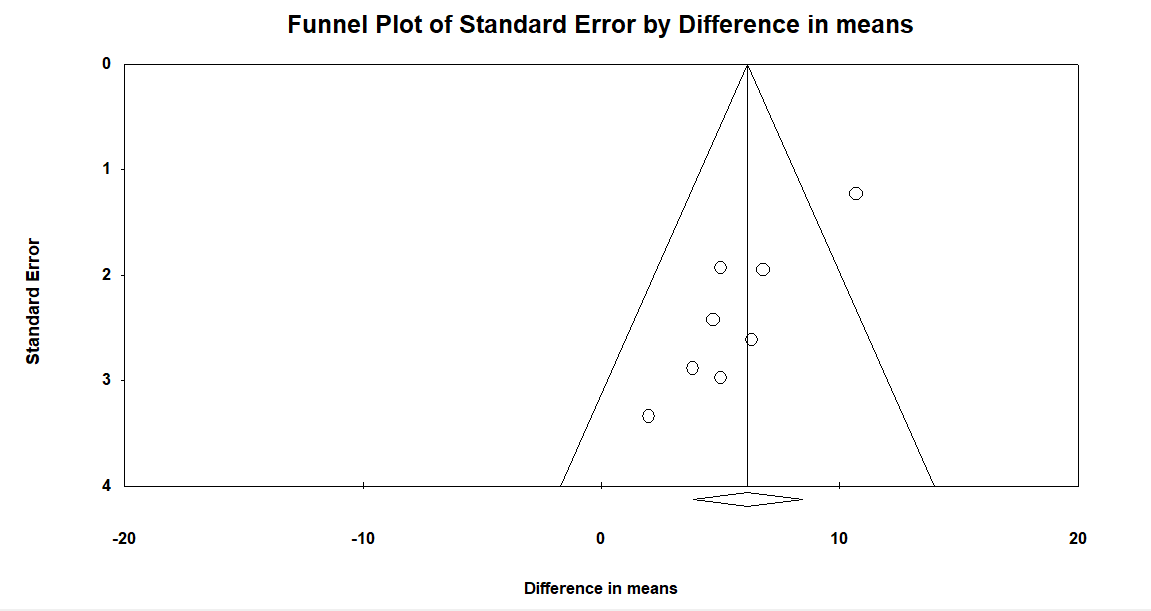
**

**Online supplementary Fig.S8:** Meta-regression

**Fig.S8a:** HF hospitalization


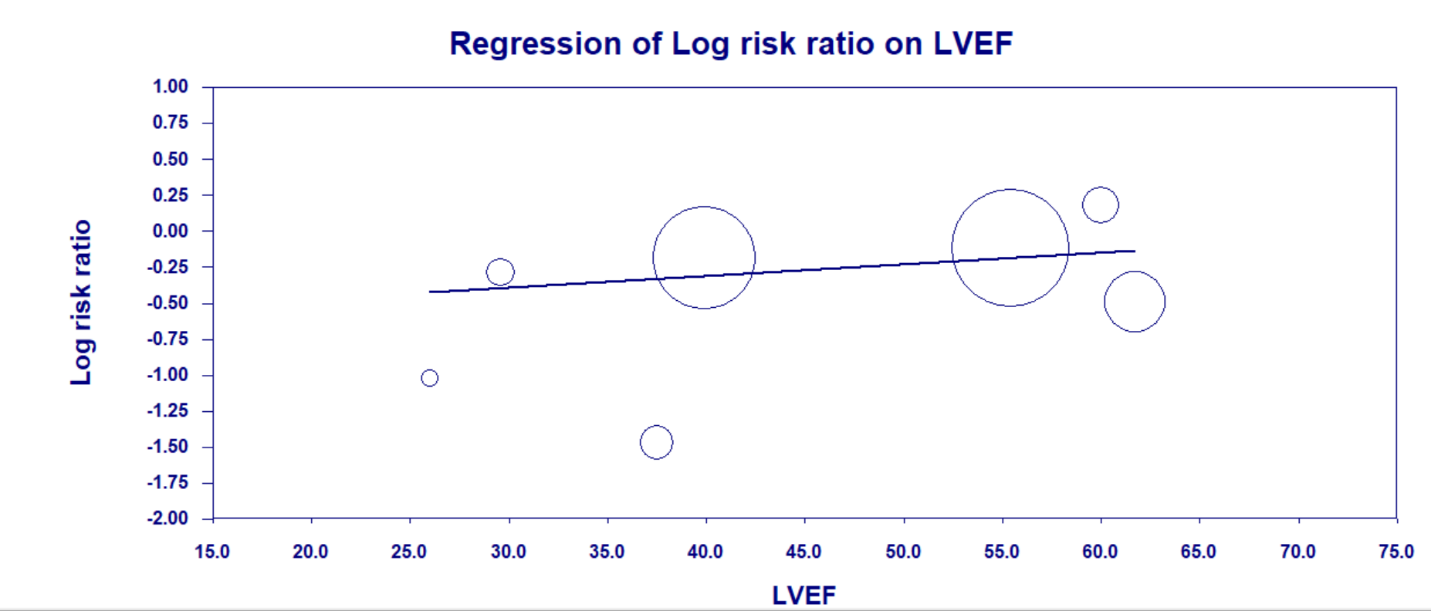


**Fig.S8b:** Mortality


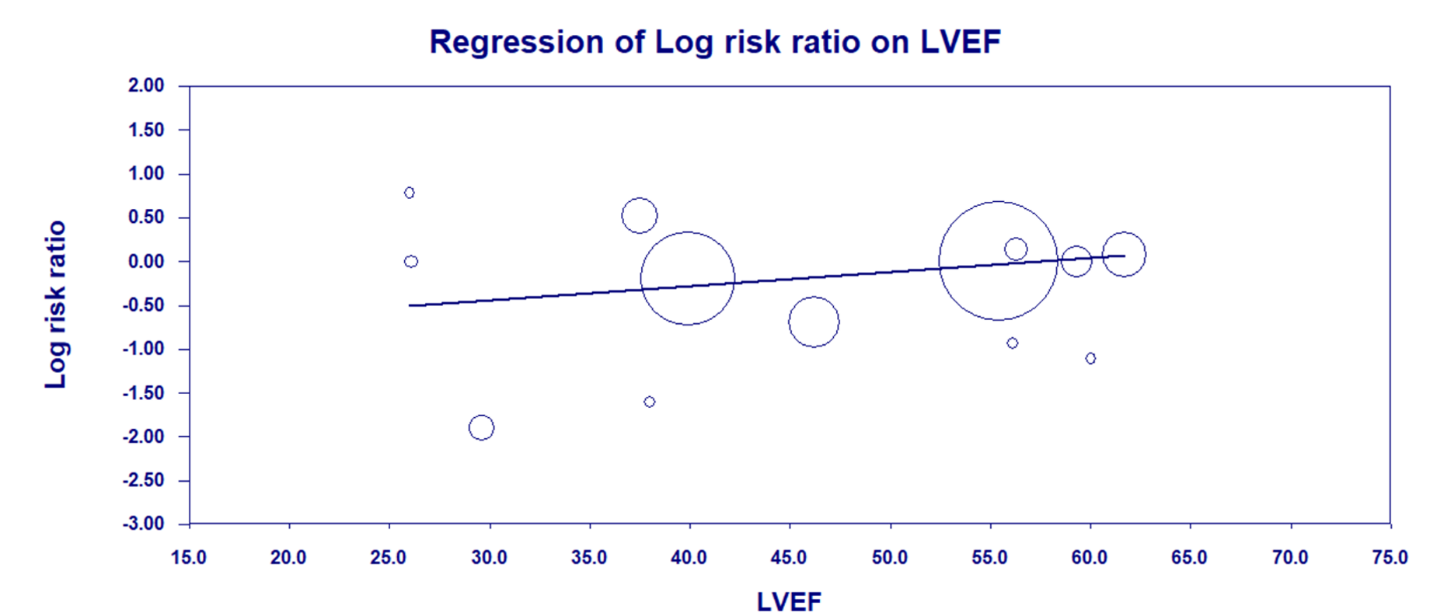

Supplement: Supplementary file 1 — Supplementary Material 1 [file 12872_2025_5336_MOESM1_ESM.docx]
